# Multiple trait multiple interval mapping of quantitative trait loci from inbred line crosses

**DOI:** 10.1186/1471-2156-13-67

**Published:** 2012-08-01

**Authors:** Luciano Da Costa E Silva, Shengchu Wang, Zhao-Bang Zeng

**Affiliations:** 1 Department of Statistics & Bioinformatics Research Center, North Carolina State University, Raleigh, NC 27695-7566, USA; 2 Department of Genetics, North Carolina State University, Raleigh, NC 27695-7566, USA

**Keywords:** Genetic architecture, Genotypic variance-covariance, Pleiotropy, Power, QTL by environment interaction, Score statistics, Statistical genetics

## Abstract

**Background:**

Although many experiments have measurements on multiple traits, most studies performed the analysis of mapping of quantitative trait loci (QTL) for each trait separately using single trait analysis. Single trait analysis does not take advantage of possible genetic and environmental correlations between traits. In this paper, we propose a novel statistical method for multiple trait multiple interval mapping (MTMIM) of QTL for inbred line crosses. We also develop a novel score-based method for estimating genome-wide significance level of putative QTL effects suitable for the MTMIM model. The MTMIM method is implemented in the freely available and widely used Windows QTL Cartographer software.

**Results:**

Throughout the paper, we provide compelling empirical evidences that: (1) the score-based threshold maintains proper type I error rate and tends to keep false discovery rate within an acceptable level; (2) the MTMIM method can deliver better parameter estimates and power than single trait multiple interval mapping method; (3) an analysis of *Drosophila* dataset illustrates how the MTMIM method can better extract information from datasets with measurements in multiple traits.

**Conclusions:**

The MTMIM method represents a convenient statistical framework to test hypotheses of pleiotropic QTL versus closely linked nonpleiotropic QTL, QTL by environment interaction, and to estimate the total genotypic variance-covariance matrix between traits and to decompose it in terms of QTL-specific variance-covariance matrices, therefore, providing more details on the genetic architecture of complex traits.

## Background

Many traits that are important to agriculture, human health and evolutionary biology are quantitative in nature, influenced by multiple genes. Efficient and robust identification and mapping onto genomic positions of those genes is a very important goal in quantitative genetics. The availability of genome-wide molecular markers provides the means for us to locate and map those quantitative trait loci (QTL) in a systematic way. Since the publication of interval mapping method for QTL genome-wide scan [1], many statistical methods have been proposed and developed to map multiple QTL with or without epistasis in single trait in a variety of populations [2], e.g. composite interval mapping (CIM) [3,4], least squares [5,6], multiple interval mapping (MIM) [7], and Bayesian interval mapping [8,9].

Although single trait QTL mapping methods have been applied in many studies to estimate the genetic basis and architecture of complex traits, these methods did not utilize the information of genetic and environmental correlations between traits, and are not ideal for data analysis. Multiple trait analysis however can take these into account and also can formally test a number of hypotheses concerning the nature of genetic correlations, such as pleiotropy vs. close linkage and genotype by environment interaction [10]. Moreover, multiple trait analysis can allow the estimation of genetic variance-covariance matrix between traits and its decomposition in terms of individual QTL ( [11,12] pages 109-110).

Multiple trait CIM [10], least squares [13] and Bayesian [14,15] methods have been available for multiple trait QTL analysis. However, these methods have not been targeted to multiple QTL for multiple traits, i.e. the whole QTL that contribute to the genetic variances and covariances. Also these methods lack appropriate criteria for assessing genome-wide significance level of QTL effects. The multiple trait CIM method uses a genome-wide threshold based on either asymptotic approximation of the log-likelihood ratio test (LRT) or permutation [16]. Nevertheless, when applied to multiple QTL models, the permutation test has some limitations in testing some targeted hypotheses. In this study, we have invested efforts in developing: (1) a statistical method for multiple trait multiple interval mapping (MTMIM) of QTL from inbred line crosses, and (2) a score-based threshold for assessing significance level of QTL that is suitable for MTMIM.

In what follows, we motivate MTMIM modeling from a practical point of view, describe the MTMIM statistical model, build the likelihood function, derive parameter estimators, extend the score-based threshold method [17] to the MTMIM model, propose a forward selection strategy to build an MTMIM model using the score-based threshold as a criterion to assess the significance level of QTL effects, and propose a model optimization procedure to fine tune a fitted MTMIM model. We then frame the hypothesis testing of pleiotropic versus closely linked non-pleiotropic QTL, and QTL by environment interaction via the MTMIM model. Next, we implement the MTMIM model and score-based threshold method and evaluate them with several simulated datasets. More specifically, we evaluate type I error, model fitting, and the efficiency of the test of pleiotropic versus closely linked nonpleiotropic QTL delivered by the MTMIM model. Lastly, we demonstrate the usefulness of the MTMIM model by analyzing data from an experiment with fruit flies *Drosophila* and draw our final considerations.

We organize this paper in a manner such that a reader less interested in the mathematical aspect of the modeling could skip the analytical derivations while being able to understand the main points regarding multiple trait multiple interval mapping of QTL.

### A motivating example

We use data from a cross between fruit flies *Drosophila simulans* and *D. mauritiana* to motivate MTMIM modeling. Detailed information about the experiment can be found in [18,19]. Briefly, males from an inbred line of *D. mauritiana* (Rob A JJ) were crossed to females from an inbred line of *D. simulans* (13w JJ) to produce F_1_ hybrids. F_1_ females were then crossed to each parental species to produce two backcross populations of males, *mauritiana* backcross (BM) and *simulans* backcross (BS). These two crosses were repeated one more time to produce two independent populations from each backcross: BS1 (sample size n=186), BS2 (n=288), BM1 (n=192) and BM2 (n=299). Males from BM1 and BS1 were scored at 45 marker loci for which the two parental lines were homozygous for different alleles. Males from BM2 and BS2 were scored at 42 marker loci out of the same 45 marker loci that BM1 and BS1 were scored. The phenotypic values of each subject are: (1) average over both sides (left and right) of the first principal component of 100 Fourier coefficients of posterior lobe (PC1); (2) area of the posterior lobe (AREA); (3) average over both sides of the first principal component of 100 Fourier coefficients of the rescaled posterior lobe, rescaled so that it has unit area (ADJPC1); and (4) length of the foreleg tibia (TIBIA). While PC1 provides a measure of both size and shape of the posterior lobe, AREA and ADJPC1, on the other hand, provide measures of size and shape somewhat separately. TIBIA provides a measure of overall body size. The genotypic and phenotypic data are freely available at [20].

All variables related to the posterior lobe (PC1, ADJPC1 and AREA) were reported to be highly correlated between themselves in both BM1 and BS1, correlation larger than 0.82 [18]. Therefore, suggesting the presence of pleiotropic and/or closely linked QTL affecting size and shape. However, all variables related to the posterior lobe were weakly correlated with TIBIA. Because posterior lobe shape and size possibly share most of their developmental process components, these two traits could be tightly related mostly due to pleiotropic effects [18]. Results of composite interval mapping analysis of AREA, PC1, and ADJPC1 were very similar to each other, except for the presence of a QTL affecting both AREA and PC1 but not ADJPC1 in the interval between marker loci *Ddc* and *eve*. Therefore, this QTL affects size but not shape of the posterior lobe [18]. In this article, we use only PC1 and ADJPC1 traits and the BM1 and BM2 samples. AREA was not analyzed because it is highly correlated (0.99) with PC1 [18], and TIBIA was not analyzed because according to Liu and coauthors [18] it has small correlation with AREA and in general TIBIA is not an important factor governing the variability of posterior lobe shape. Besides, on our single trait analysis no QTL was found for TIBIA. BS1 and BS2 samples were not used for analysis because the main goal of this article is to present a novel method for QTL mapping, rather than to investigate details of the inheritance of posterior lobe shape.

**Figure 1:**
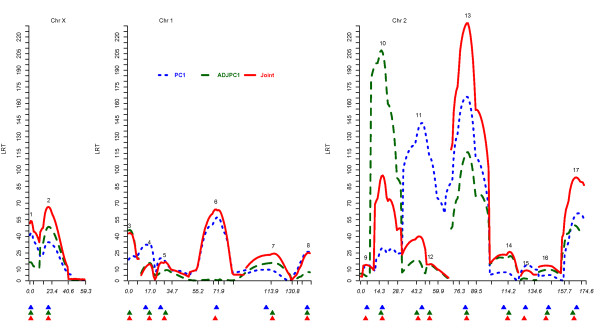
**LRT profile of separate MIM analyses of PC1 and ADJPC1, and MTMIM analysis of PC1 and ADJPC1 (Joint) for the BM data.** LRT profile of separate MIM analyses of PC1 and ADJPC1, and MTMIM analysis of PC1 and ADJPC1 (Joint) for the BM data with 10% genome-wide significance level. Tick marks in the horizontal axis represent positions of genetic markers on chromosomes X, 2 and 3 (from left to right). Bold triagles bellow the horizontal axis indicate positions of mapped QTL in separate and joint analyses. Map distances are expressed in centiMorgans according to Haldane’s mapping function.

We carried out MIM analysis of PC1 and ADJPC1 in the pooled samples of BM1 and BM2 (n=192+299), hearafter referred as BM data, and we found statistical evidence for seventeen genomic regions harboring QTL (Figure 1), of which twelve genomic regions showed statistical evidence of QTL affecting both traits (perhaps pleiotropic QTL), and five regions showed statistical evidence of QTL affecting either one of the traits (regions 3, 6, 9 , 12 and 15). We want to mention that in all these five regions, expect region 6, even for the trait for which the effect is not statistically significant there is still some evidence of weak putative QTL effect, as shown in the LRT profiles from the MIM analysis of PC1 and ADJPC1. Region 6, which includes marker loci *Ddc* and *eve*, was previously reported not to harbor any putative QTL with significant effect on ADJPC1 [18]. Overall, the inferred genomic regions harboring putative QTL in our MIM analysis are in strong agreement with previous inferred QTL in [18,19].

Positions of mapped QTL in regions 4, 5, 7, 10, 11, 13, 14, 16 and 17 (Figure 1) did not coincide in the MIM models of PC1 and ADJPC1. Therefore, one could hypothesize the existence of two closely linked nonpleiotropic QTL at each of these regions. In order to test the hypotheses of pleiotropic versus closely linked nonpleiotropic QTL at each one of these regions, a joint analysis of PC1 and ADJPC1 is needed. The joint analysis also allows us to partition the genotypic variance-covariance matrix between traits PC1 and ADJPC1 in terms of QTL-specific variance-covariance matrices. Thus in this motivating example, the main reasons to use the MTMIM model are: (1) to test pleiotropic versus closely linked nonpleiotropic QTL, and (2) to estimate the contribution of each QTL to the total genotypic variance-covariance matrix between traits PC1 and ADJPC1. A third reason for the MTMIM modeling, though not applicable to this specific motivating data, is the possibility to test the hypothesis of QTL by environment interaction [10].

## Results and discussion

### Type I error

**Figure 2:**
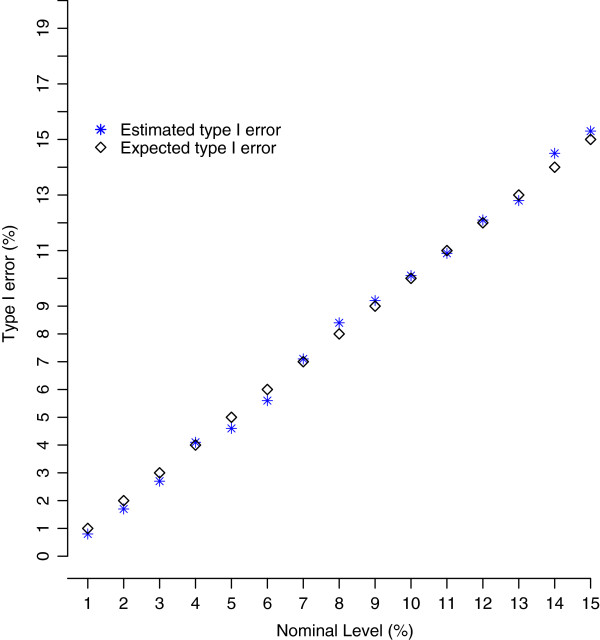
**Estimated and expected genome-wide type I error.** Estimated and expected type I error, in percentage, of LRT when using the genome-wide score-based threshold to assess significance level of putative QTL in genome-wide scan of 1000 replicates.

The results show clearly an excellent agreement between estimated type I error and nominal level in the range of 1 to 15% (Figure 2).

### Model size (results not shown)

The number of QTL in the MTMIM model of scenario SI was much closer to the simulated parameter (five QTL) when compared to scenario SII, for any genome-wide significance level. While a QTL in both scenarios has to exceed very similar thresholds to be declared significant in the forward selection, the number of traits affected by a QTL is rather different between the two scenarios. In scenario SI all QTL have effect on all traits, while in scenario SII a QTL may have effect either on one, two or three traits. Therefore, model overparametrization makes the detection of QTL with effects on one and two traits in scenario SII more difficult. Lastly, our results show that in general the number of mapped QTL is closer to the simulated (five QTL) in the MTMIM than in the MIM model.

### FDR

FDR is a very import measure of quality control in statistical analysis [21]. However, FDR is not feasibly estimated in analysis of data from traditional QTL experiments, due to the low discovery rate of putative QTL in such experiments. Nevertheless, in simulation experiments we are able to estimate FDR because we can replicate the experiment many times. We estimate FDR (Table 1) when varying the genome-wide significance levels (1, 5, and 10%) and LOD-*d* support interval levels (*d*=1, 1.5 and 2). While FDR is expected to increase with increments in genome-wide significance level, our results show that for a fixed LOD-*d* level FDR changed little with increments in genome-wide significance levels, in both MIM and MTMIM models. Regarding changes in LOD-*d* level, our results show that FDR and LOD-*d* are negatively correlated, as expected. Higher levels of LOD-*d* ultimately translate into wider LOD-*d* support intervals, therefore, increasing chances of capturing the true position of QTL. FDR in the MIM and MTMIM models were very similar, except in the MIM model of trait T3 of scenario SII, which was simulated with only one QTL of small effect (heritability of 5%).

**Table 1:** Estimates of false discovery rate

**Analysis**		**SI**			**SII**			**SIII**		
**(trait)**	**LOD-*d***	**1%**	**5%**	**10%**	**1%**	**5%**	**10%**	**1%**	**5%**	**10%**
MIM	1.0	9.1	9.1	9.9	8.9	9.2	10.0	7.2	7.9	8.7
(T1)	1.5	3.9	4.4	5.3	3.7	4.3	5.3	2.8	3.5	4.1
	2.0	2.0	2.7	3.6	1.8	2.2	3.0	1.4	1.9	2.3
MIM	1.0	8.0	8.7	8.9	7.9	8.6	9.6	6.2	7.0	7.8
(T2)	1.5	3.9	4.2	4.7	3.2	4.1	5.4	3.1	3.7	4.5
	2.0	2.0	2.3	3.0	1.2	2.2	3.6	1.2	2.1	2.8
MIM	1.0	10.7	9.6	9.9	12.4	13.8	18.0	–	–	–
(T3)	1.5	3.8	4.2	4.9	7.5	9.0	11.4	–	–	–
	2.0	1.8	2.3	3.1	4.8	6.5	8.5	–	–	–
MTMIM	1.0	4.6	5.4	6.9	8.5	9.2	10.0	5.6	7.8	8.4
	1.5	1.9	2.7	4.0	3.3	4.1	4.9	2.9	5.2	5.7
	2.0	1.1	1.9	3.3	1.4	2.4	3.2	2.2	4.1	4.5

Estimates of FDR (%) in the MIM and MTMIM models as observed in scenarios SI, SII and SIII across genome-wide significance levels (1, 5 and 10%) and LOD-*d* support intervals.

### Power

Results of power for the MIM and MTMIM models of all three scenarios clearly show a remarkable increment in power as genome-wide significance levels grow less stringent, for any LOD-*d* level (Table 2 - results shown for LOD-1.5 level only). Based on these results as well as on those that showed almost constance of FDR across genome-wide significance levels, we, hereafter, show and discuss results of 10% genome-wide significance level only.

**Table 2:** Power of QTL identification

**Analysis**		**SI**			**SII**			**SIII**		
**(trait)**	**QTL**	**1%**	**5%**	**10%**	**1%**	**5%**	**10%**	**1%**	**5%**	**10%**
	Q1	66.8	82.0	86.6	65.8	80.2	84.2	67.6	77.2	79.6
MIM	Q2	63.6	81.8	87.6	59.8	78.2	81.8	–	–	–
(T1)	Q3	67.4	81.6	87.2	63.2	81.2	85.8	75.2	87.0	90.2
	Q4	66.4	81.8	87.0	63.4	78.4	83.4	–	–	–
	Q5	66.8	83.6	86.4	65.6	82.0	87.2	70.2	78.4	81.6
	Q1	64.8	80.0	88.2	–	–	–	–	–	–
MIM	Q2	64.8	80.0	84.8	74.4	85.4	89.8	64.2	74.2	76.4
(T2)	Q3	65.6	79.8	83.4	76.4	86.0	90.0	76.4	88.4	91.2
	Q4	66.0	82.4	87.0	77.4	87.6	92.0	74.6	86.0	88.0
	Q5	68.4	83.0	88.8	–	–	–	–	–	–
	Q1	65.6	81.4	86.0	–	–	–	–	–	–
MIM	Q2	63.2	80.0	86.6	–	–	–	–	–	–
(T3)	Q3	65.6	80.4	84.0	53.4	70.6	77.8	–	–	–
	Q4	65.4	80.8	87.8	–	–	–	–	–	–
	Q5	65.4	83.0	88.6	–	–	–	–	–	–
	Q1	98.8	99.4	99.4	53.8	71.0	78.2	65.4	65.2	70.0
MTMIM	Q2	98.0	98.0	98.2	89.0	94.4	95.6	64.6	66.6	68.0
	Q3	97.0	97.4	97.4	96.6	97.0	97.2	94.4	96.4	97.0
	Q4	98.4	98.8	99.0	87.6	93.2	94.6	74.8	77.4	78.2
	Q5	98.6	98.6	98.6	57.2	71.8	78.4	65.6	66.2	68.0

Power (%) of QTL identification in the MIM and MTMIM models as observed in scenarios SI, SII and SIII across genome-wide significance levels (1, 5, and 10%) and LOD-1.5 support interval.

Results of power (10% genome-wide significance level and LOD-1.5) to identify QTL in the MTMIM model show that QTL affecting more traits have higher chances of being identified in the forward selection. In scenario SI, which is the most favorable among all three scenarios, all QTL have effects on all traits. Therefore, all QTL were correctly identified very often, power ≥ 97*%* (Table 2). In scenario SII, Q1 has effect on one trait only, Q2 on two traits, and Q3 on three traits. Power increases from Q1 (78.2%) to Q3 (97.2%) in the MTMIM model. Results also show that the MTMIM model can have lower power to identify QTL that has effects on only a small subset of traits when compared to the MIM model, due to greater genome-wide threshold in the MTMIM model. For instance, MTMIM model has less power (78.2%) than MIM model (84.2%) to identify Q1, which affects only T1 (same pattern is seen for Q5). However, as the subset of traits affected by a QTL increases, power of MTMIM model overpasses power of MIM model, even when some traits are not affected by that QTL. For instance, Q2 affects T1 and T2, but not T3, nevertheless, MTMIM model identifies Q2 (95.2%) more frequently than MIM model (81.8%) (same pattern carries over to Q4). The increment in power as the number of traits affected by a QTL increases was also observed in scenario SIII.

In scenarios SII and SIII, we decomposed power of QTL identification (10% genome-wide significance level and LOD-1.5) into three nonoverlapping subsets (Table 3). In scenario SII, there is a subset of replicates for which a QTL affects T1 only, another subset for which a QTL affects T1 and T2 simultaneously, and finally a subset of replicates for which a QTL affects all traits (T1, T2, and T3) simultaneously. In scenario SIII, there is a subset of replicates for which a QTL affects T1 only, another subset for which a QTL effects T2 only, and finally a subset of replicates for which a QTL affects T1 and T2 simultaneously. These decompositions of power allow us to decompose the total power in the MTMIM model into QTL-trait power, therefore enabling us to measure the frequency in which a nonpleiotropic QTL is mapped as a pleiotropic one. In scenario SII, where all QTL are independent, most of power to identify a QTL is concentrated on the simulated trait affected by that QTL. For instance, in the LOD-1.5 level, 66.4 out of 78.2% power (0.85 ratio) to identify Q1 is due to T1 alone, which is the only trait in which Q1 has effect on. In scenario SIII, because linkage between QTL pairs Q1 and Q2, and Q4 and Q5, the contribution of simulated traits affected by these QTL to their overall power is lower than in scenario SII, though the simulated traits still account for a large amount of power. For example, 36.8 out of 70% power (0.53 ratio) to identify Q1 is due to T1 alone, which is the only trait in which Q1 has effect on, and 46 out 68% (0.68 ratio) power to identify Q5 is due to T1 alone, which is the only trait in which Q5 has effect on. Notice that in scenario SIII Q1 was mapped as a pleiotropic QTL (subset (1,1) in Table 3) more often than Q5, i.e. 30.4 out 70% (0.43 ratio) and 20.8 out of 68% (0.31 ratio), respectively. Identification of Q1 as being pleiotropic more often than Q5 is mainly because the distance between Q1 and Q2 is shorter than the distance between Q4 and Q5, 10 and 15 cM, respectively. The smaller the distance between two nonpleiotropic QTL, the harder is to separate them in the MTMIM model. Moreover, separation of nonpleiotropic QTL is also affected by the distance between genetic markers. Linkage maps with markers closely spaced are expected to help in separating nonpleiotropic QTL. On the other hand, separation of nonpleiotropic QTL in linkage maps with sparse markers, such as the linkage map used in our simulations, is a much harder task.

**Table 3:** Decomposition of total power into QTL-trait power

**Scenario**		**Subsets**														
		**(1,0,0)**					**(1,1,0)**					**(1,1,1)**				
		**Q1**	**Q2**	**Q3**	**Q4**	**Q5**	**Q1**	**Q2**	**Q3**	**Q4**	**Q5**	**Q1**	**Q2**	**Q3**	**Q4**	**Q5**
SII	P_*trait*_	66.4	1.2	0.0	0.8	64.0	4.2	86.4	5.0	87.2	8.2	0.8	6.6	89.0	5.8	0.2
	ratio	0.85	0.01	0.00	0.01	0.82	0.05	0.90	0.05	0.92	0.10	0.01	0.07	0.92	0.06	0.00
		**(1,0)**					**(0,1)**					**(1,1)**				
SIII	P_*trait*_	36.8	2.8	3.4	1.0	46.0	2.8	36.2	4.0	49.6	1.2	30.4	29.0	89.6	27.6	20.8
	ratio	0.53	0.04	0.04	0.01	0.68	0.04	0.53	0.04	0.63	0.02	0.43	0.43	0.92	0.35	0.31

Decomposition of total power (P_*total*_ in Table 2) from scenarios SII and SIII into QTL-trait power (P_*trait*_) with 10% genome-wide significance level and LOD-1.5 support interval. In SII, subsets (1, 0, 0), (1, 1, 0) and (1, 1, 1) contain replicates with QTL affecting T1 only, T1 and T2, and T1, T2 and T3, respectively. In SIII, subsets (1, 0), (0, 1) and (1, 1) contain replicates with QTL affecting T1 only, T2 only, and T1 and T2, respectively. The QTL-trait to the overall power ratio (ratio=P_*trait*_ /P_*total*_) is also presented.

### Mean position of QTL

Our simulations show that mean estimates of QTL position in the MIM and MTMIM models have no qualitative difference and are in close agreement with the simulated parameters (Table 4). There is, though, a trend of smaller variation (measured in terms of standard error of mean) in the MTMIM than in the MIM model. Also, in the MTMIM model there is a trend of smaller variation for those QTL with effects on a larger subset of traits.

**Table 4:** Means of QTL position, LOD-*d* support interval coverage and length

			**Position**			**Coverage**		**Length**	
**Analysis (Trait)**	**QTL**	**Parameter**	**Estimate**	**1**	**1.5**	**2**	**1**	**1.5**	**2**
MIM (T1)	Q1	23 [1]	23.7 (0.31)	91.4	95.7	99.3	21.7 (0.42)	29.4 (0.55)	37.3 (0.66)
	Q2	15 [2]	14.6 (0.31)	92.2	95.8	98.1	21.1 (0.38)	27.7 (0.55)	34.9 (0.73)
	Q3	45 [3]	45.4 (0.38)	88.8	95.8	98.2	23.7 (0.49)	33.0 (0.67)	41.9 (0.81)
	Q4	67 [5]	66.9 (0.29)	92.2	95.8	98.4	20.2 (0.35)	26.7 (0.51)	35.4 (0.79)
	Q5	53 [6]	52.9 (0.33)	93.4	98.8	99.6	21.3 (0.43)	28.7 (0.56)	36.4 (0.68)
MIM (T2)	Q2	15 [2]	14.7 (0.30)	92.6	97.4	98.7	21.0 (0.88)	27.9 (0.55)	34.1 (0.67)
	Q3	45 [3]	45.2 (0.35)	90.6	95.9	98.3	22.3 (0.38)	29.8 (0.56)	39.1 (0.74)
	Q4	67 [5]	67.0 (0.27)	95.3	98.1	99.6	19.6 (0.33)	26.1 (0.49)	32.6 (0.67)
MIM (T3)	Q3	45 [3]	44.7 (0.45)	88.8	94.6	96.8	25.3 (0.55)	35.3 (0.74)	46.2 (0.88)
MTMIM	Q1	23 [1]	23.5 (0.32)	89.5	95.6	97.6	20.0 (0.38)	26.4 (0.47)	33.1 (0.56)
	Q2	15 [2]	14.4 (0.22)	93.1	97.8	98.9	16.2 (0.25)	21.0 (0.33)	25.3 (0.39)
	Q3	45 [3]	44.9 (0.18)	92.8	97.2	99.4	13.1 (0.22)	17.2 (0.28)	20.7 (0.33)
	Q4	67 [5]	67.6 (0.19)	94.2	97.5	98.9	15.6 (0.23)	20.3 (0.31)	24.2 (0.39)
	Q5	53 [6]	52.8 (0.37)	89.5	97.8	99.8	19.7 (0.41)	26.1 (0.51)	32.6 (0.60)

Means of QTL position (cM), LOD-*d* support interval coverage (%) and length (cM) in the MIM and MTMIM models as observed in scenario SII across LOD-*d* support intervals (1, 1.5 and 2) and 10% genome-wide significance level. Position estimates shown here are for the LOD-1.5 support interval only. The chromosome in which each QTL is located is shown between square brackets. Standard errors of means are between parentheses.

### Coverage and length of LOD-d support interval

In Table 4, we show the results of coverage and length of LOD-d support interval, and as can be seen, coverage for any LOD-*d* level are not remarkably different between the MIM and MTMIM models. However, on average the estimates of LOD-*d* support interval length were always larger in the MIM model. Differences in length are only marginal for QTL with effects on only a small subset of traits, but there are considerable differences for those QTL with effects on larger subset of traits. For instance, in scenario SII Q1 affects one trait only and it has LOD-1.5 support interval mean length of 29.4 cM in the MIM and 26.4 cM in the MTMIM model. On the other hand, Q2 affects two traits and it has LOD-1.5 support interval mean length of 27.7 (T1) and 27.9 (T2) in the MIM models and 21.0 cM in the MTMIM model. An interesting result is that the LOD-1.5 support interval produced confidence intervals with approximately 95% coverage in both MIM and MTMIM models.

### Mean effect of QTL

The average of effects of QTL in scenario SI (Table 5) shows that estimates of QTL effects in the MTMIM model are overall in close agreement with the simulated parameters, mostly because of high power in this scenario. Results of scenario SII demonstrate the robustness of the MTMIM model in estimating the effects of QTL, whereby QTL without effects on certain traits have estimates near zero, while QTL with nonzero effects have estimates with low bias. However, the robustness of the MTMIM to estimate QTL effect with low bias is less evident in scenario SIII. For instance, notice that while Q2 has zero effect on T1, its effect estimate is not close to zero. In order to understand why this bias is present in Q2 of scenario SIII, we need to understand how we matched a mapped to a simulated QTL. In the forward selection we searched and mapped pleiotropic QTL, then each mapped pleiotropic QTL was tested against the alternative hypothesis of closely linked nonpleiotropic QTL at the neighboring region of the mapped pleiotropic QTL. If the pleiotropic hypothesis was not rejected, we assumed the QTL was pleiotropic. Then, in order to apply our summary statistics, each mapped pleiotropic QTL was matched to its closest (smallest distance) simulated QTL. It could happen that a mapped pleiotropic QTL in the neighboring region of simulated Q1 and Q2 be matched to Q2, even though the major effect of the mapped pleiotropic QTL comes from Q1. Notice that when the previous situation happens, we mistakenly assign the effect of Q1 (which affects only T1) to Q2 (which presumably would not affect T1), therefore, producing biased estimated effect of Q2 on T1. The same explanation of “bias” carries over to Q4 (T1), Q1 (T2) and Q5 (T2) in scenario SIII. We quoted bias to emphasize that the bias observed in scenario SIII is not due to the MTMIM estimation *per se*, but rather due to our lack of ability to separate closely linked nonpleiotropic QTL or due to our criterion to match mapped to simulated QTL.

**Table 5:** Mean effect of QTL

			**SI**		**SII**		**SIII**	
**Trait**	**QTL**	**Parameter**	**MIM**	**MTMIM**	**MIM**	**MTMIM**	**MIM**	**MTMIM**
T1	Q1	0.52	0.57 (0.006)	0.51 (0.007)	0.56 (0.005)	0.56 (0.005)	0.57 (0.006)	0.56 (0.011)
	Q2	0.52	0.56 (0.006)	0.51 (0.006)	0.56 (0.006)	0.52 (0.007)	–	0.20 (0.019)
	Q3	0.52	0.56 (0.006)	0.52 (0.006)	0.54 (0.005)	0.51 (0.007)	0.57 (0.005)	0.52 (0.008)
	Q4	0.52	0.55 (0.006)	0.51 (0.006)	0.55 (0.006)	0.52 (0.006)	–	0.13 (0.015)
	Q5	0.52	0.56 (0.006)	0.52 (0.007)	0.55 (0.006)	0.56 (0.005)	0.58 (0.005)	0.58 (0.013)
T2	Q1	0.52	0.55 (0.007)	0.50 (0.007)	–	0.00 (0.004)	–	0.23 (0.016)
	Q2	0.52	0.56 (0.005)	0.51 (0.006)	0.57 (0.006)	0.54 (0.007)	0.58 (0.006)	0.55 (0.009)
	Q3	0.52	0.56 (0.005)	0.52 (0.006)	0.57 (0.005)	0.54 (0.007)	0.57 (0.005)	0.54 (0.008)
	Q4	0.52	0.55 (0.005)	0.50 (0.006)	0.57 (0.005)	0.55 (0.006)	0.58 (0.006)	0.60 (0.008)
	Q5	0.52	0.55 (0.006)	0.52 (0.007)	–	0.00 (0.005)	–	0.09 (0.015)
T3	Q1	0.52	0.56 (0.005)	0.52 (0.006)	–	0.00 (0.005)	–	–
	Q2	0.52	0.55 (0.005)	0.51 (0.007)	–	0.01 (0.004)	–	–
	Q3	0.52	0.55 (0.005)	0.51 (0.006)	0.51 (0.006)	0.44 (0.008)	–	–
	Q4	0.52	0.55 (0.005)	0.52 (0.007)	–	0.00 (0.003)	–	–
	Q5	0.52	0.56 (0.006)	0.53 (0.008)	–	0.00 (0.004)	–	–

Mean effect of QTL in the MIM and MTMIM models as observed in scenarios SI, SII and SIII with 10% genome-wide significance level and LOD-1.5 support interval. Standard errors of means are between parentheses.

The effects of all QTL were overestimated in the MIM model. This phenomena is expected due to estimation conditional on detection, the so-called “Beavis effect” [22]. A qualitative comparison of results show that overall the estimation of QTL effects in the MTMIM model are less biased than in the MIM model.

### Pleiotropic versus closely linked nonpleiotropic QTL

In scenario SIII, after selecting an MTMIM model in the forward selection, each mapped pleiotropic QTL was tested against the alternative of closely linked nonpleiotropic QTL. In the bivariate model, we performed a two-dimensional search for positions of putative closely linked nonpleiotropic QTL in the neighborhood of the position of each pleiotropic QTL, as suggested in [10]. The model with nonpleiotropic QTL that showed highest likelihood within the two-dimensional search region was selected and tested against the model with pleiotropic QTL. We compared two criteria for model selection, the AICc and LRT. The critical value for the LRT at 5% significance level was obtained from a chi-squared probability distribution with one degree of freedom.

Because Q3 was simulated as being pleiotropic, rejection of pleiotropic hypothesis for Q3 provides a measure of type I error. On the other hand, Q1 and Q2, and Q4 and Q5 were simulated as pairs of closely linked nonpleiotropic QTL. Therefore, rejection of pleiotropic hypothesis at these QTL provides a measure of power. Under our simulation setting, the LRT performed better than the AICc. The LRT was able to keep the best balance between type I error and power. Estimated frequency of rejecting pleiotropy for Q3 (4%) using the LRT agrees very well with the expected 5% nominal error rate, and estimated frequency of rejecting pleiotropy for Q1 (38%) and Q2 (36%) are satisfactory high, taking into account that Q1 and Q2 are considerably close to each other in a linkage map with markers considerably distant from each other (10 cM from marker-to-marker). On the other hand, the AICc criterion showed higher power for Q1 (45%) and Q2 (45%), but with a cost of high type I error for Q3 (15%). Moreover, because Q4 and Q5 are 15 cM apart from each other, the frequency of rejecting pleiotropy using LRT for these two QTL (41 and 48%, respectively) is higher than for Q1 (38%) and Q2 (36%), which are 10 cM apart from each other.

### Motivating example revisited

Motivated by the fact that the joint analysis of PC1 and ADJPC1 in the *Drosophila* dataset could provide additional information to distinguish between genetic effects of QTL on size and shape of posterior lobe, we then analyzed these two traits with the MTMIM model. Such additional information are: (1) testing pleiotropic versus closely linked nonpleiotropic QTL, and (2) estimating the contribution of each QTL in the fitted model to the genotypic variance-covariance matrix between PC1 and ADJPC1. In what follows, we show results of the MIM and MTMIM model of the pooled samples from BM1 and BM2 (n=192+299), the BM data. We also take advantage of this dataset to test the GEM-NR algorithm for maximizing the likelihood function under the MTMIM model with many QTL. Using data from a genetic experiment would provide more realistic comparisons between the GEM-NR and ECM algorithms than a simulated dataset would do.

The LRT profiles of genome-wide scan in the BM data (Figure 1) shows that the MTMIM model produced smaller values of LRT than the MIM model for some genomic positions, therefore, seemingly violating the expectation that the MTMIM model would produce greater LRT values than the nested MIM models [10]. Nevertheless, this violation is easily explained because not all positions of putative QTL in the MIM and MTMIM models coincide. Therefore, the MIM models are not nested within the MTMIM model shown here. Seventeen regions in the genome showed statistical evidence of putative QTL in the MTMIM model with 10% genome-wide significance level (Figure 1 and Table 6).

**Table 6:** Estimates of QTL position and main effect on PC1 and ADJPC1 of BM data

	**MIM**				**MTMIM (GEM-NR)**			**MTMIM (ECM)**	
	**PC1**		**ADJPC1**		**(PC1 and ADJPC1)**			**(PC1 and ADJPC1)**	
**QTL**	$\hat{p}$^***a***^	${\hat{\beta }}_{1}$	$\hat{p}$	${\hat{\beta }}_{2}$	$\hat{p}$	${\hat{\beta }}_{1}$	${\hat{\beta }}_{2}$	${\hat{\beta }}_{1}$	${\hat{\beta }}_{2}$
Chromosome X									
1	1	0.0020	1	0.0165	1	0.0021	0.0175	0.0021	0.0175
2	20	0.0018	20	0.0284	20	0.0017	0.0275	0.0017	0.0275
Chromosome 2									
3	–	–	1	0.0304	1	0.0007	0.0293	0.0007	0.0293
4	14	0.0018	17	0.0215	17	0.0018	0.0220	0.0018	0.0220
5	26	0.0017	30	0.0141	29	0.0012	0.0146	0.0011	0.0146
6	71	0.0016	–	–	70	0.0017	-0.0048^*ns*^	0.0017	-0.0048^*ns*^
7	111	0.0009	116	0.0147	116	0.0011	0.0176	0.0011	0.0177
8	144	0.0012	144	0.0091	144	0.0011	0.0082	0.0011	0.0082
Chromosome 3									
9	5	0.0013	–	–	4	0.0011	0.0107	0.0011	0.0107
10	17	0.0022	16	0.0503	17	0.0022	0.0427	0.0022	0.0426
11	48	0.0033	44	0.0279	45	0.0027	0.0253	0.0027	0.0254
12	–	–	54	0.0235	54	0.0007^*ns*^	0.0255	0.0007	0.0254
13	82	0.0033	83	0.0391	83	0.0034	0.0394	0.0034	0.0394
14	112	0.0009	116	0.0324	115	0.0009	0.0257	0.0009	0.0257
15	129	0.0015	–	–	128	0.0012	0.0094^*ns*^	0.0012	0.0094^*ns*^
16	147	0.0007	146	0.0116	145	0.0009	0.0092	0.0009	0.0092
17	169	0.0021	166	0.0268	167	0.0021	0.0273	0.0021	0.0273
Total QTL	15		14		17				
${\hat{\sum }}_{p}$						2.761	31.73		
						31.73	521.6		
${\hat{\sum }}_{g}$	2.358		–			2.369	31.48		
	–		453.0			31.48	453.2		

Estimates of QTL position ( $\hat{p}$) and main effect on PC1 ( ${\hat{\beta }}_{1}$) and ADJPC1 ( ${\hat{\beta }}_{2}$) in the MIM and MTMIM models of BM data with 10% genome-wide significance level. QTL effects in the MTMIM model were estimated via GEM-NR and ECM algorithms. Estimated phenotypic ( ${\hat{\sum }}_{p}$) and genotypic ( ${\hat{\sum }}_{g}$) variance-covariance matrices (multiplied by 10^5^ ) are also shown.

^*a*^ Estimated position (cM) of QTL from the leftmost genetic marker on the chromosome.

^*ns*^ Nonsignificant main effect tested with the LRT and 5% significance level. The critical value of the LRT was obtained from the chi-squared distribution function with one degree of freedom.

MIM models of PC1 and ADJPC1 all together showed statistical evidence of twelve genomic regions with statistical significant QTL affecting both traits, and five regions with statistically significant QTL affecting either one of the traits (regions 3, 6, 9 , 12 and 15 shown in Figure 1 and Table 6). MTMIM model mapped these five regions either exactly or very close to their respective estimated positions in the MIM models. Moreover, the estimated effects of these five regions in the MTMIM model showed small discrepancy from those estimates in the MIM models (Table 6). Nevertheless, empirical results from our simulations suggest that both estimates of positions and effects of QTL in the MTMIM model are more accurate than in the MIM models.

Positions of QTL in regions 4, 5, 7, 10, 11, 13, 14, 16 and 17 (Figure 1 and Table 6) did not coincide with those in the MIM models of PC1 and ADJPC1. Therefore, one could hypothesize the existence of two closely linked nonpleiotropic QTL at each of these regions. We tested the hypothesis of pleiotropic QTL versus closely linked nonpleiotropic QTL at each of these regions, and on the basis of the data available the null hypothesis of pleiotropic QTL could not be rejected for any region. Thus, since PC1 contains attributes of both shape and size of posterior lobe, whereas ADJPC1 contains attributes of size only, the available data provides strong evidence that the genetic mechanisms controlling shape and size of posterior lobe are highly similar.

Partition of the phenotypic variance-covariance matrix between PC1 and ADJPC1 in terms of their environmental and genotypic components, as estimated in the MTMIM model, shows that most of the phenotypic variance-covariance between these traits is due to the genotypic component (Table 6). Moreover, we partitioned the total genotypic variance-covariance matrix in terms of QTL-specific variance-covariance matrices (Table 7) as proposed in [11] and [12] (pages 109-110). This decomposition of the genotypic variance-covariance matrix shows how much of the total genotypic variance-covariance is explained by each QTL in the fitted model.

**Table 7:** Estimated QTL-specific (multiplied by 10^5^) genotypic variance-covariance matrix between traits PC1 and ADJPC1

		**QTL**																																	
**Traits**	**QTL**	**1**		**2**		**3**		**4**		**5**		**6**		**7**		**8**		**9**		**10**		**11**		**12**		**13**		**14**		**15**		**16**		**17**	
PC1	1	0.11	0.93	0.12	1.49	0.00	0.08	0.01	0.07	0.00	0.03	0.00	-0.01	0.00	0.05	0.01	0.07	0.00	-0.02	-0.01	-0.11	-0.03	-0.28	-0.01	-0.13	0.01	0.06	-0.01	-0.10	-0.01	-0.05	0.00	0.02	0.00	0.03
ADJ		0.93	7.69	1.49	16.20	0.08	1.06	0.07	0.64	0.03	0.30	-0.01	0.11	0.05	0.57	0.07	0.54	-0.02	-0.16	-0.11	-1.32	-0.28	-2.44	-0.13	-1.74	0.06	0.61	-0.10	-1.23	-0.05	-0.39	0.02	0.21	0.03	0.28
PC1	2			0.08	1.21	0.00	0.00	0.00	0.00	0.00	0.02	0.00	-0.03	0.01	0.10	0.01	0.09	0.00	0.02	0.00	0.05	-0.01	-0.16	0.00	-0.05	0.01	0.20	-0.01	-0.13	-0.01	-0.10	0.00	0.00	-0.01	-0.10
ADJ				1.21	19.13	0.00	-0.05	0.00	0.05	0.02	0.26	-0.03	0.17	0.10	1.57	0.09	0.92	0.02	0.29	0.05	0.81	-0.16	-1.83	-0.05	-1.09	0.20	2.66	-0.13	-2.57	-0.10	-1.00	0.00	0.04	-0.10	-1.36
PC1	3					0.01	0.52	0.05	1.30	0.02	0.47	0.00	0.09	0.00	-0.03	0.00	-0.04	0.00	0.07	0.00	-0.02	-0.01	-0.22	0.00	-0.08	-0.01	-0.28	0.00	-0.01	0.00	0.04	0.00	0.04	0.00	-0.04
ADJ						0.52	21.64	1.30	24.16	0.47	9.37	0.09	-0.57	-0.03	-0.63	-0.04	-0.51	0.07	1.06	-0.02	-0.55	-0.22	-3.36	-0.08	-3.13	-0.28	-5.17	-0.01	-0.20	0.04	0.49	0.04	0.70	-0.04	-0.72
PC1	4							0.10	1.18	0.07	0.92	0.03	0.15	0.00	0.00	-0.01	-0.05	0.01	0.11	0.00	-0.02	-0.03	-0.32	-0.01	-0.16	-0.02	-0.24	0.01	0.13	0.01	0.07	0.01	0.06	0.00	-0.03
ADJ								1.18	14.08	0.92	11.44	0.15	-1.12	0.00	-0.03	-0.05	-0.45	0.11	1.14	-0.02	-0.36	-0.32	-3.32	-0.16	-2.94	-0.24	-2.88	0.13	2.22	0.07	0.64	0.06	0.68	-0.03	-0.41
PC1	5									0.02	0.31	0.03	0.13	0.00	0.04	0.00	-0.01	0.00	0.05	0.00	-0.02	-0.01	-0.13	0.00	-0.07	-0.01	-0.10	0.00	0.05	0.00	0.03	0.00	0.03	0.00	0.03
ADJ										0.31	4.06	0.13	-0.93	0.04	0.57	-0.01	-0.05	0.05	0.50	-0.02	-0.39	-0.13	-1.40	-0.07	-1.44	-0.10	-1.19	0.05	0.83	0.03	0.32	0.03	0.37	0.03	0.38
PC1	6											0.07	-0.20	0.02	0.16	0.01	0.02	0.00	-0.01	-0.01	-0.06	-0.01	-0.02	0.00	-0.01	0.00	0.00	0.00	0.05	0.00	0.01	0.00	0.01	0.00	0.01
ADJ												-0.20	0.57	0.16	-1.08	0.02	-0.17	-0.01	0.08	-0.06	0.39	-0.02	0.16	-0.01	0.08	0.00	0.03	0.05	-0.33	0.01	-0.05	0.01	-0.07	0.01	-0.09
PC1	7													0.03	0.49	0.03	0.32	0.00	0.01	0.00	0.08	0.00	0.03	0.00	0.02	0.00	0.02	0.00	-0.10	0.00	-0.05	0.00	-0.03	-0.01	-0.11
ADJ														0.49	7.76	0.32	3.09	0.01	0.09	0.08	1.38	0.03	0.30	0.02	0.35	0.02	0.21	-0.10	-2.07	-0.05	-0.56	-0.03	-0.41	-0.11	-1.61
PC1	8															0.03	0.22	0.00	0.00	0.00	0.04	0.00	-0.02	0.00	0.00	0.00	0.03	0.00	-0.04	0.00	-0.01	0.00	-0.01	0.00	-0.02
ADJ																0.22	1.54	0.00	0.01	0.04	0.37	-0.02	-0.13	0.00	0.01	0.03	0.26	-0.04	-0.44	-0.01	-0.11	-0.01	-0.04	-0.02	-0.15
	9																	0.03	0.30	0.08	1.24	0.04	0.34	0.01	0.15	-0.01	-0.08	0.00	0.01	0.00	0.00	0.00	0.00	0.00	0.00
																		0.30	2.88	1.24	16.13	0.34	3.25	0.15	2.34	-0.08	-0.85	0.01	0.14	0.00	0.00	0.00	-0.01	0.00	-0.05
	10																			0.12	2.32	0.13	1.93	0.02	0.73	0.02	0.29	0.00	0.06	0.00	-0.01	0.00	-0.04	-0.01	-0.20
																				2.32	45.50	1.93	24.29	0.73	18.94	0.29	4.25	0.06	1.36	-0.01	-0.07	-0.04	-0.61	-0.20	-3.05
	11																					0.18	1.64	0.07	1.66	0.15	1.58	0.01	0.27	0.01	0.08	0.00	-0.01	-0.01	-0.14
																						1.64	15.19	1.66	24.92	1.58	16.29	0.27	3.81	0.08	0.68	-0.01	-0.14	-0.14	-1.48
	12																							0.01	0.38	0.05	1.16	0.00	0.14	0.00	0.07	0.00	0.00	0.00	-0.07
																								0.38	14.74	1.16	20.87	0.14	4.55	0.07	0.89	0.00	-0.04	-0.07	-1.41
	13																									0.27	3.12	0.05	0.91	0.04	0.39	0.01	0.17	0.01	0.09
																										3.12	36.41	0.91	15.12	0.39	3.66	0.17	1.89	0.09	1.06
	14																											0.02	0.53	0.04	0.70	0.02	0.31	0.01	0.31
																												0.53	15.11	0.70	8.66	0.31	5.01	0.31	5.53
	15																													0.04	0.29	0.03	0.30	0.04	0.38
																														0.29	2.27	0.30	2.82	0.38	3.73
	16																															0.02	0.18	0.05	0.57
																																0.18	2.03	0.57	6.83
	17																																	0.11	1.44
																																		1.44	18.55
	Total																																	2.36	31.48
																																		31.48	453.20

**Figure 3:**
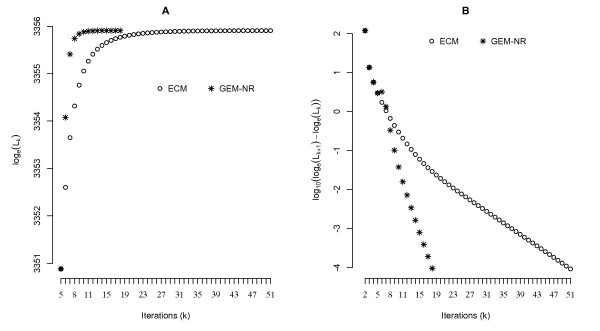
**Comparison of performances between ECM and GEM-NR algorithms.** Comparison of performances between ECM and GEM-NR algorithms in terms of number of iterations required to the convergence of the likelihood function. Both algorithms were applied to an MTMIM model of traits PC1 and ADJPC1 of the BM data. The algorithms were said to have converged whenever the difference between the natural logarithm of the likelihood function of two consecutive iterations was smaller than or equal to 10^−4^. (**A**) shows the values of the natural logarithm of the likelihood function at each iteration [log_*e*_ (L_*k*_)] until convergence was reached. The GEM-NR algorithm began with 5 iterations of ECM algorithm. Therefore, the first 5 iterations produced identical values in the likelihood function of both algorithms, and because of that we omitted the first 4 iterations. (**B**) shows the difference between the natural logarithm of the likelihood function of two consecutive iterations until convergence was reached. In (**B**), the y-axis was rescaled via logarithm of base ten to improve graphical resolution.

The possibility of fitting many traits and many QTL in the MTMIM model imposes severe burden in the estimation of parameters both in terms of reliability of parameter estimates (accuracy) and computation time (speed). The GEM-NR and ECM algorithms are two alternative approaches suitable for parameter estimation in such complex models. We evaluate these two algorithms with the BM data by fitting an MTMIM model for PC1 and ADJPC1. The results (Figure 3) show a tremendous gain of GEM-NR over ECM in terms of number of iterations, 19 and 52, respectively, as well as in terms of computing time, 8.2 and 30.6 seconds in a desktop PC, respectively. The gain in computation time from GEM-NR is even more evident in genome-wide scan and model selection because likelihood maximization have to be computed many times. Parameter estimates delivered in the GEM-NR and ECM were very similar (Table 6).

## Conclusions

A novel statistical method for multiple trait multiple interval mapping (MTMIM) of QTL from inbred line crosses was proposed and developed. We also proposed a novel method for estimating genome-wide threshold and assessing the significance level of putative QTL effects in the MTMIM model. The method of genome-wide threshold estimation is based on the score-based resampling framework [17]. The MTMIM model has the advantage of allowing us to map QTL with effects on multiple traits, while taking advantage of information from correlations between traits. The MTMIM model has been implemented in the freely available software Windows QTL Cartographer [23].

The MTMIM model provides a comprehensive framework for QTL inference on multiple traits and the score-based threshold serves as an essential and elegant tool for computing significance level of effects of putative QTL in the genome-wide scan. The MTMIM model and score-based threshold were evaluated through simulations. Also, we analyzed data from an experiment with *Drosophila* for the purpose of illustrating the MTMIM model and evaluating the performances of the GEM-NR and ECM algorithms.

Results from our simulations showed many interesting features of the MTMIM model and score-based threshold. First, the score-based threshold maintained the type I error at a desired nominal level when no QTL effects were present in the simulated datasets. Second, discovery of spurious QTL (false discovery rate) was almost constant across genome-wide significance levels of 1, 5 and 10%, while power to identify simulated QTL increased substantially as the significance level grew less stringent. Therefore, a more liberal (10%) genome-wide significance level could be used in the genome-wide scan, corroborating the results of C. Laurie, S. Wang, L. A. Carlini-Garcia and Z-B. Zeng as observed in the MIM model (unpublished observations). Third, the MTMIM model could show lower power than the MIM model for QTL with effects on only a small subset of traits. However, as the number of traits affected by a QTL increases, power in the MTMIM model overpasses power in the MIM model even when not all traits under analysis are affected by that QTL. Forth, on average the estimates of QTL position in the MIM and MTMIM models were very similar, but the MTMIM model delivers estimates with smaller sampling variances. Fifth, the LOD-1.5 support interval produced confidence intervals for QTL position with approximately 95% coverage in both the MIM and MTMIM models. However, the support interval was much wider in the MIM than in MTMIM model. Overall, a qualitative comparison of results from the MIM and MTMIM models shows that effect estimates in the latter are less biased than in the former. Lastly, the LRT was shown to keep adequate type I error level when testing the null hypothesis of pleiotropic QTL against the alternative of closely linked nonpleiotropic QTL in the bivariate analysis, while it delivered reasonable power when data were generated under the alternative.

Throughout this paper, we provided compelling empirical evidences that the score-based threshold maintained proper type I error rate and tend to give a false discovery rate within acceptable level, and that the MTMIM model can deliver better parameter estimates and power than the MIM model, and yet the MTMIM model provides a framework to test hypotheses of pleiotropic QTL versus closely linked nonpleiotropic QTL, QTL by environment interaction, and to estimate the total genotypic variance-covariances matrix between traits and to decompose it in terms of QTL-specific variance-covariance matrices. An analysis of phenotypic and genotypic data from an experiment with *Drosophila* illustrated the new tools present in the MTMIM model. In conclusion, the MTMIM model is a valuable tool to better extract information from experiments with measurements in multiple quantitative traits, therefore, providing more details on the genetic architecture of complex traits.

## Methods

In what follows, for any matrix ***A***, its transpose is denoted by $A^{\prime }$, its inverse by ***A***^−1^, its *u*^*t*ℎ^ row by ***A***_[*u*,·]_, its *v*^*t*ℎ^ column by ***A***_[·,*v*]_, and its element in row *u* and column *v* by ***A***_[*u*,*v*]_ .

### Statistical model

Our statistical model for multiple trait multiple QTL inference for a backcross (BC) population is a linear model, in which the measurement *y*_*ti*_ of trait *T* (*T* = 1,2,· · ·, *T*) on each subject *i* (*i* = 1,2,· · · *n*) is regressed on variables *x*_*ir*_ (*r* = 1,2,· · · *m*). These variables are defined according to Cockerham genetic model [24,25]. For each subject *i*, *x*_*ir*_ takes either value $\frac{1}{2}$ or $-\frac{1}{2}$, depending on whether QTL *r* has homozygous or heterozygous genotype, respectively. The coefficient *β*_*tr*_ is called the main effect of the *r*^*t*ℎ^ QTL on trait *T*. The linear model also includes an intercept *u*_*T*_ for each trait, it may include a subset *p* of epistatic effects (*w*_*trl*_) among all pairwise QTL interactions (*r* and *l* ∈ {1,2,· · · *m*}), and it includes a residue *e*_*ti*_ . The linear model is:

(1) $$y_{\mathrm{ti}}=u_{t}+\overset{m}{\underset{r=1}{\sum }}\beta_{\mathrm{tr}}x_{\mathrm{ir}}+\overset{p}{\underset{r<l}{\sum }}w_{\text{trl}}x_{\mathrm{ir}}x_{\mathrm{il}}+e_{\mathrm{ti}}$$

For each subject *i*, let $y_{i}={\left(y_{1i},y_{2i},\cdot \cdot \cdot \;,y_{\mathrm{Ti}}\right)}^{\prime }$ be a *T* × 1 vector of trait measurements, and $e_{i}={\left(e_{1i},e_{2i},\cdot \cdot \cdot \;,e_{\mathrm{Ti}}\right)}^{\prime }$ be a *T* × 1 random vector assumed to be independent and identically distributed according to a multivariate normal distribution with mean vector zero and positive definite symmetric variance-covariance matrix **∑**_*e*_, i.e., ***e***_*i*_ ∼ *MVN*_*T*_ (**0,∑**_*e*_). For each *r*, let $\beta_{r}={\left(\beta_{1r},\beta_{2r},\cdot \cdot \cdot \;,\beta_{\mathrm{Tr}}\right)}^{\prime }$ be a column vector of main effects. For each pair *r* and *l* (*r* < *l*, *r* = 1,2,· · · ,*p*) of interaction, let $w_{b}={\left(w_{1\mathrm{rl}},w_{2\mathrm{rl}},\cdot \cdot \cdot \;,w_{\mathrm{Trl}}\right)}^{\prime }$ be a column vector of epistatic effects (*b* = 1,2,· · · ,*p*). Lastly, let $u={\left(u_{1},u_{2},\cdot \cdot \cdot \;,u_{T}\right)}^{\prime }$ be a *T* × 1 vector of means.

We collect all effect parameters (*m* main and *p* epistatic effect vectors) into a *T* × *s* (*s* = *m* + *p*) matrix $B\;=\;(\;\beta_{1},\;\beta_{2},\;\cdot \cdot \cdot \;,\;\beta_{m},\;w_{1},\;w_{2},\;\cdot \cdot \cdot \;,\;w_{p})$, and all model parameters into a column vector ***θ*** = (***θ***_1_, ***θ***_2_,· · ·, ${\theta_{s},\mu^{\prime },\text{vect}(\sum_{e}))}^{\prime }$, where $\theta_{b}=\beta_{b}^{\prime }$ for 1 ≤ * b* ≤ *m* and $\theta_{b}=w_{b}^{\prime }$ for *m* <*b* ≤ *s*, and *vect*(***∑***_*e*_) is an operator that stacks the rows of ***∑***_*e*_ into a column vector one on the top of the other and then transposes it. Motivated by the fact that a QTL may not have significant effect on all traits under analysis, we allow for the insignificant parameter effects in each vector ***θ***_*b*_ to be constrained to zero. Therefore, the MTMIM model allows each trait to have its own set of effect parameters, as in the *seemingly unrelated regression* model [26].

### Likelihood function

In order to search the entire genome for significant QTL effects, the genome is partitioned into *H* points, usually at 1-centiMorgan (cM) grid. This partition is denoted by ***ζ***. The set of positions of *m* putative QTL, ***λ*** = {*λ*_1_*λ*_2_,· · · *λ*_*m*_ }, is assumed to be a subset of ***ζ***[27]. For any subject *i*, let *M*_*i*_ be the genotypic information of markers flanking the *m* QTL, and $M_{i,L}^{r}$ and $M_{i,R}^{r}$ be the flanking markers on left and right of QTL *r*, respectively. In a diploid species, a subject from a BC population generated from inbred line crosses has either genotype *QQ* or *Qq* for a locus, assuming the recurrent parent has genotype QQ. Therefore, if there are *m* QTL affecting a trait, there are 2^*m*^ possible genotypes for any subject *i*. Genotypes of the form *G*_*j*_ = _*Q*1_*Q*_2_ · · ·*Q*_*m*_, where *Q*_*r*_ ∈ {*QQQq*}, *r* = 1,2,· · · *m* and *j* = 1,2,· · · 2^*m*^. Then, assuming no crossover interference between marker intervals and no more than one QTL existing within a marker interval, the probability of any genotype *G*_*j*_, conditional on the genotypes of markers flanking the *m* QTL is $p_{\mathrm{ij}}=P(G_{j}|M_{i},R,\lambda )=\overset{m}{\underset{r=1}{\prod }}P\left(Q_{r}\left(\right|M_{i,L}^{r},M_{i,R}^{r},\lambda_{r}\right)$, where the probabilities on the right hand side of this equation can be estimated via a Hidden Markov model [28].

We define an *s* × 2^*m*^ matrix *Z* of coded genotypes according to Cockerham genetic model [24,25]. In the matrix *Z* each row *b*, *Z*_[*b*,·]_, corresponds to a column of effect parameters in B(b=1,2,· · ·,s) and each column *j*, *Z*_[·,*j*]_, represents a coded genotype *G*_*j*_ . If *b* ≤ *m*, *Z*_[*b*,*j*]_ = *x*_*r*_, otherwise *Z*_[*b*,*j*]_ = *x*_*r*_ ∗*x*_*l*_, where *x*_*u*_ (*u* ∈ {*r*, *l*}) is either $\frac{1}{2}$ or $-\frac{1}{2}$, depending on whether the genotype of QTL *Q*_*u*_ in *G*_*j*_ is *QQ* or *Qq*, respectively.

The individual (*L*_*i*_) and overall likelihood (*L*) functions of data under the MTMIM model with *m* QTL are mixtures of 2^*m*^ multivariate normal distribution functions with different means ( $u+BZ_{\left[\cdot ,j\right]}$), assumed same variance-covariance (**∑**_*e*_), and mixing probabilities *p*_*ij*_ (*j* = 1,2,· · · ,2^*m*^), i.e., $L_{i}(\theta |y_{i},M_{i},\lambda )=\overset{2^{m}}{\underset{j=1}{\sum }}p_{\mathrm{ij}}\phi (y_{i}|u+BZ_{\left[\cdot ,j\right]},\sum_{e})$ and $L(\theta |Y,M,\lambda )=\overset{n}{\underset{i=1}{\prod }}L_{i}(\theta |y_{i},M_{i},\lambda )$, where *y* is a *T* × *n* matrix of trait measurements, and $\phi (y_{i}|u+BZ_{\left[\cdot ,j\right]},\sum_{e})$ is the probability distribution function of a multivariate normal random variable *y*_*i*_ with mean $u+BZ_{\left[\cdot ,j\right]}$ and variance-covariance ***∑***_*e*_ . In what follows, *ℓ*_*i*_ (***θ***|***y***_*i*_,_*Mi*_,***λ***) and *ℓ*(***θ***|*y*,*M*,***λ***) are the natural logarithm of the individual and overall likelihood functions, respectively.

### Parameter estimation

Estimation of parameters in the likelihood function is cumbersome due to mixture of distributions. The expectation-maximization (EM) [29] algorithm is very popular for parameter estimation in mixture models. The EM algorithm is very simple to program, given that efficient estimators are available for the “complete-data”. Moreover, the EM algorithm guarantees that the likelihood function is nondecreasing in every iteration. However, EM may show slow convergence rate if there are many missing data, and EM does not provide standard errors of parameter estimates.

Many modifications of the EM algorithm and many hybrids of EM and Gauss-Newton (GN) methods have been proposed [30-32]. GN methods are not guaranteed to converge when the logarithm likelihood function is not concave, but if there is convergence its rate is usually quadratic, as opposite to the linear rate of EM. Therefore, speed of convergence of GN may be much faster than EM. We describe two algorithms to obtain the maximum likelihood estimators (MLE) of parameters in the MTMIM model: expectation-conditional maximization (ECM) and a hybrid of EM and Newton-Raphson called generalized EM-NR (GEM-NR).

#### Expectation-conditional maximization algorithm

The EM algorithm [29] solves the incomplete logarithm likelihood function iteratively in terms of the unobserved complete-data logarithm likelihood function. If the complete-data logarithm likelihood function is messy and the M-step is complex, then the EM algorithm is no longer attractive. For such cases of complicate M-step, [33] proposed a class of generalized EM algorithm, called expectation-conditional maximization (ECM). The ECM enjoys the convergence properties of the EM while simplifying the estimation of parameters. In the ECM, a complex M-step is broken down into many simpler CM-steps, each one of them maximizes the expected complete-data logarithm likelihood function conditional on some function of the parameters. Besides simplifying the M-step, the CM-step is often faster and more stable than the M-step because the conditional maximization are over spaces of smaller dimensions [33].

*E-step*: The E-step requires computation of the expectation of the complete-data logarithm likelihood function, conditional on the observed data *y* and evaluated at a current value of ***θ*** (see Appendix). The E-step at the (*v* + 1) iteration consists of updating the probabilities Π_*ij*_ as follows:

$$\Pi_{\mathrm{ij}}^{\left(\nu +1\right)}=\frac{p_{\mathrm{ij}}\phi \left(y_{i}|\mu^{\left(\nu \right)}+B^{\left(\nu \right)}Z_{\left[\cdot ,j\right]},\sum_{e}^{\left(\nu \right)}\right)}{\overset{2^{m}}{\underset{j=1}{\sum }}p_{\mathrm{ij}}\phi \left(y_{i}|\mu^{\left(\nu \right)}+B^{\left(\nu \right)}Z_{\left[\cdot ,j\right]},\sum_{e}^{\left(\nu \right)}\right)}$$

It is worth mentioning that in the E-step above, the updating equation at step *v* + 1 does not use the probabilities from the previous step *v*, i.e, it uses *p*_*ij*_ instead of $\Pi_{\mathrm{ij}}^{\left(\nu \right)}$. This is the case in QTL mapping literature because the *a priori* probabilities are indeed exellent estimates of the conditional probabilities of QTL given the flanking markers.

The CM-step consists of maximizing the expected complete logarithm likelihood function with respect to the unknown parameters (see Appendix). *CM-step without constrained parameters*: We split the parameters into the groups $B_{\left[\cdot ,1\right]},B_{\left[\cdot ,2\right]},\cdot \cdot \cdot \;,B_{\left[\cdot ,s\right]}$, ***u***, and **∑**_*e*_ . Parameters within the same group are estimated simultaneously, while parameters in distinct groups are estimated consecutively. The parameter estimators can be shown to be:

$$\;\begin{array}{c} \mu^{\left(\nu +1\right)}=\frac{1}{n}\overset{n}{\underset{i=1}{\sum }}\left(y_{i}-\overset{2^{m}}{\underset{j=1}{\sum }}\Pi_{\mathrm{ij}}^{\left(\nu +1\right)}B^{\left(\nu \right)}Z_{\left[\cdot ,j\right]}\right)\\ \sum_{e}^{\left(\nu +1\right)}=\frac{1}{n}\overset{n}{\underset{i=1}{\sum }}\overset{2^{m}}{\underset{j=1}{\sum }}\Pi_{{}_{\mathrm{ij}}}^{\left(\nu +1\right)}\;\left(y_{i}\;-\;\mu^{\left(\nu +1\right)}\;-\;B^{\left(\nu \right)}Z_{\left[\cdot ,j\right]}\right)\;{\left(\;y_{i}\;-\;\mu^{\left(\nu +1\right)}\;-\;B^{\left(\nu \right)}Z_{\left[\cdot ,j\right]}\;\right)}^{\prime }\\ B_{\left[\cdot ,b\right]}^{\left(\nu +1\right)}=\frac{\overset{n}{\underset{i=1}{\sum }}\overset{2^{m}}{\underset{j=1}{\sum }}\Pi_{\mathrm{ij}}^{\left(\nu +1\right)}\;\;\left(\;y_{i}\;-\;\mu^{\left(\nu +1\right)}\;-\;\;\overset{b-1}{\underset{u=1}{\sum }}\;B_{\left[\cdot ,u\right]}^{\left(\nu +1\right)}Z_{\left[u,j\right]}\;-\;\;\overset{s}{\underset{u=b+1}{\sum }}\;\;B_{\left[\cdot ,u\right]}^{\left(\nu \right)}Z_{\left[u,j\right]}\;\;\right)\;Z_{\left[b,j\right]}}{\overset{n}{\underset{i=1}{\sum }}\overset{2^{m}}{\underset{j=1}{\sum }}\Pi_{\mathrm{ij}}^{\left(\nu +1\right)}Z_{\left[b,j\right]}^{2}} \end{array}$$

for *b* ∈ {1,2,· · · ,*s*}.

*CM-step with constrained parameters*: The estimator of $B_{\left[\cdot ,b\right]}$ shown previously is not appropriate if some parameters in $B_{\left[\cdot ,b\right]}$ are constrained to zero. For instance, when estimating parameters in a model with closely linked nonpleiotropic QTL. If there exist zero-constrained effect parameters in the MTMIM model, our strategy is to update each element in $B_{\left[\cdot ,b\right]}$ one at a time. Given the current estimate $B_{\left[\cdot ,b\right]}^{\left(\nu \right)}$, the updating equation for the unconstrained effect parameter $B_{\left[t,b\right]}$ is:

$$\;\begin{array}{c} B_{\left[t,b\right]}^{\left(\nu +1\right)}\;\;=\;\frac{\overset{n}{\underset{i=1}{\sum }}\;\overset{2^{m}}{\underset{j=1}{\sum }}\Pi_{\mathrm{ij}}^{\left(\nu +1\right)}\;\sum_{e_{{}_{\left[t,\cdot \right]}}}^{-1^{\left(\nu \right)}}\;\;\left[\;\;\left(y_{i}\;-\;\mu^{\left(\nu \right)}\;\right)\;-\;\;\overset{b-1}{\underset{u=1}{\sum }}\;B_{\left[\cdot ,u\right]}^{\left(\nu +1\right)}Z_{\left[u,j\right]}\;-\;\;\;\;\;\overset{s}{\underset{u=b+1}{\sum }}\;\;\;\;B_{\left[\cdot ,u\right]}^{\left(\nu \right)}Z_{\left[u,j\right]}\;\;\right]\;\;Z_{\left[b,j\right]}}{\overset{n}{\underset{i=1}{\sum }}\overset{2^{m}}{\underset{j=1}{\sum }}\Pi_{\mathrm{ij}}^{\left(\nu +1\right)}\sum_{e_{{}_{\left[t,t\right]}}}^{-1^{\left(\nu \right)}}Z_{\left[b,j\right]}^{2}} \end{array}$$

The E- and CM-steps are computed iteratively until convergence of the likelihood function. Our choice of initial values for ***u*** and **∑**_*e*_ are the sample mean and the sample variance-covariance, respectively, and all parameters in B  are set to zero. In the genome-wide scan, an alternative efficient choice of initial values is to use converged parameters of a previous position in the search grid. For any small positive real number *ε*, a stoping rule for the convergence of the likelihood function can be defined as [*L*(***θ***^(*v* + 1)^|*Y*, *M*, ***λ***)−*L*(***θ***^(*v*)^|*Y*, *M*, ***λ***)] / *L*(***θ***^(*v*)^|*Y*, *M*, ***λ***) < *ε*.

It is worth mentioning that for many combinations of *i* and *j*, the probabilities *p*_*ij*_ are zero or very close to zero. Therefore, one may choose to ignore unimportant small probabilities in the computations, which may lead to significant improvement on computation time.

#### Generalized EM algorithm based on Newton-Raphson methods

The generalized EM-Newton-Raphson (GEM-NR) methods combine the EM algorithm with the NR method for maximizing the complete-data logarithm likelihood function [30,31]. The hybrid methods take advantage of the EM algorithm for generating an accurate starting point for the NR algorithm, which usually has faster convergency rate. By introducing a step-size *κ*^(*v*)^ (0 <*κ*^(*v*)^ ≤ 1) and by having the incomplete-data logarithm likelihood function (*ℓ*) replaced by the expected complete-data logarithm likelihood function (*Q*_*c*_) in the updating NR formula, a modified version of the updating equation [32] (see Appendix) is:

(2) $$\;\theta^{\left(\nu +1\right)}=\theta^{\left(\nu \right)}+\kappa^{\left(\nu \right)}\;{\left(\;{\left(-\frac{\partial^{2}Q_{c}(\theta |Y)}{\partial \theta \partial \theta^{\prime }}\right|}_{\theta^{\left(\nu \right)}}\;\right)}^{-1}\;{\left(\frac{\partial Q_{c}(\theta |Y)}{\partial \theta }\right|}_{\theta^{\left(\nu \right)}}$$

The advantage of using equation (2) is that an appropriate choice of *κ*^(*v*)^ guarantees that the logarithm likelihood function increases at each iteration. So long as *κ*^(*v*)^ is chosen to make (3) positive definite, the logarithm likelihood function is guaranteed to increase at every iteration (Appendix).

(3) $$B=\left(I+\frac{1}{2}\kappa^{\left(\nu \right)}{\left(\frac{\partial^{2}Q_{c}(\theta |Y)}{\partial \theta \partial \theta^{\prime }}\right|}_{\theta^{\left(\xi \right)}}C^{\prime }C\right)$$

where ***C*** is the Cholesky decomposition of the negative of the matrix of second order derivatives of the complete logarithm likelihood function (see Appendix) and ***I*** is an identity matrix.

To guarantee that the logarithm likelihood function is nondecreasing, [31] proposed to start the EM algorithm with five iterations to quickly approach the MLE and then to switch to NR until either convergence or decrease of the logarithm likelihood function. If the logarithm likelihood function decreases, they suggested halving the step-size *κ* up to five times. If the logarithm likelihood function still decreases, they suggested to return to the EM, run five iterations, and then to switch back to NR. [31] argued that their choice of running the EM algorithm for five iterations is based on previous experiences of [34] that 95% of the change in the initial value of logarithm likelihood function until its maximum value often happens in five EM iterations.

As ***θ***^(*ξ*)^ lies in the line segment from ***θ***^(*v*)^ to ***θ***^(*v* + 1)^, and ***θ*** lives in high-dimensional space, the choice of *κ*^(*v*)^ to make (3) positive definite may not be easy. We implemented an iterative GEM-NR procedure as follows: 

1. Run the ECM algorithm a couple of iterations (say five iterations);

2. Let ***θ***^(*v*)^ be the parameter estimate in the *v*^*t*ℎ^ EM iteration;

3. Set *κ*^(*v*)^ = 1;

4. Estimate ***θ***^(*v* + 1)^ using equation (2) with the first and second order derivatives of *Q*_*c*_ (***θ***|*y*) evaluated at ***θ***^(*v*)^ ;

5. ● If *ℓ*(***θ***^(*v* + 1)^|*Y*, *M*, ***λ***) > *ℓ*(***θ***^(*v*)^|*Y*, *M*, ***λ***), then set ***θ***^(*v* + 1)^ as the updated parameter;

  ● Otherwise, keep repeating step 4 with smaller and smaller *κ*^(*v*)^, until the likelihood function increases or until *κ*^(*v*)^ gets too small, in which case start again in step 1;

In cases in which the complete-data logarithm likelihood function does not allow for closed form solution of parameter estimators, [30] have found that the GEM-NR can reduce significantly the computation burden when compared to the EM algorithm. In the Appendix, we derived all expressions (first and second order derivatives of the complete-data logarithm likelihood function) to implement the GEM-NR algorithm.

### Genome-wide significance level and model selection

#### Score-based threshold

We extend the score statistic [17] to assess the genome-wide statistical significance level of QTL effect in the MTMIM model. Based on the individual and overall likelihood functions, we derived all required expressions to compute the score statistic to test any effect parameter in the MTMIM model (see Appendix).

Under some regular conditions, the score and LRT statistics are asymptotically equivalent in large sample [35]. But, an interesting characteristic of the score statistic is that it can be approximated by a sum of independent random components. Motivated by this characteristic and based on the decomposition of the score function [17,36] derived the large-sample distribution of the score statistic for genome-wide QTL mapping.

In multiple trait genome-wide scan, a putative pleiotropic QTL is assumed at every position *λ* ∈ ***ζ*** and the significance level of its effects (main or epistatic effects) is tested against the null of no effects. For instance, assume a model with *m* − 1 QTL with main effects and *p* epistatic effects between certain QTL pairs. Assume we are scanning for a putative *m*^*t*ℎ^ QTL. Let *l* = *λ* denotes the testing position of the putative QTL coming into the model. Let ***λ*** = (*λ*_1_,*λ*_2_,· · · ,*λ*_(*m*−1)_, *l*) be the current positions of all *m* QTL in the model. Let $\theta_{m}=\beta_{m}^{\prime }$ be a *T* × 1 vector of effects for the new QTL coming into the model, and let $\theta ={\left(\theta_{1},\theta_{2},\cdot \cdot \cdot \;,\theta_{m-1},\theta_{m},\theta_{m+1},\cdot \cdot \cdot \;,\theta_{s},u^{\prime },\text{vect}(\sum_{e})\right)}^{\prime }$ be a column vector of all parameters in the model, where $\theta_{b}=\beta_{b}^{\prime }$ for 1 ≤ *b* ≤ *m* and $\theta_{b}=w_{b}^{\prime }$ for *m* <*b* ≤ *s*. Let $\eta ={\left(\theta_{1},\theta_{2},\cdot \cdot \cdot \;,\theta_{m-1},\theta_{m+1},\cdot \cdot \cdot \;,\theta_{s},u^{\prime },\text{vect}(\sum_{e})\right)}^{\prime }$ be the column vector of nuisance parameters. Then the hypothesis H_0_ : ***θ***_*m*_ = ***0 *** versus H_1_ : ***θ***_*m*_ ≠ ***0*** is assessed at every position *l* in the genome by the LRT. The genomic position with the maximum LRT among all *l* is assessed for the presence of a QTL via the score-based method.

The score statistic to test H_0_ vs H_1_ can be written as $S=\;{\hat{U}}^{\prime }{\hat{V}}^{-1}Û$[17,36], where $Û=\overset{n}{\underset{i=1}{\sum }}{Û}_{i}$, $\hat{V}=\overset{n}{\underset{i=1}{\sum }}{Û}_{i}{Û}_{i}^{\prime }$, and ${Û}_{i}$ is:

(4) $$\begin{array}{cc} \;{Û}_{i} & ={\left(\frac{\partial \ell_{i}\left(\theta_{1},\eta \right)}{\partial \theta_{1}^{\prime }}\right|}_{\left(\theta_{1}=\theta_{10},\eta =\tilde{\eta }\right)}\\ & \;-{\left(\frac{\partial \ell \left(\theta_{1},\eta \right)}{\partial \theta_{1}\partial \eta^{\prime }}\right|}_{\left(\theta_{1}=\theta_{10},\eta =\tilde{\eta }\right)}\\ & \;\times \;{\left(\;{\left(\frac{\partial \ell \left(\theta_{1},\eta \;\right)\;}{\partial \eta \partial \eta^{\prime }}\right|}_{\left(\theta_{1}=\theta_{10},\eta =\tilde{\eta }\right)}\;\right)}^{-1}\;{\left(\frac{\partial \ell_{i}\left(\theta_{1},\eta \right)}{\partial \eta^{\prime }}\right|}_{\left(\theta_{1}=\theta_{10},\eta =\tilde{\eta }\right)} \end{array}$$

where $\tilde{\eta }$ is the MLE of ***η*** under H_0_ (see Appendix for a detailed derivation of first and second order derivatives of the likelihood function).

In order to maintain equal expected variances in the resampled score and score statistic [17], we multiply ${Û}_{i}$ by random variables *z*_*i*_ from the univariate normal distribution with mean zero and unit variance, i.e. *z*_*i*_ ∼ *N*(0, 1). Let ${Û}_{i}(l)$ be equation (4) evaluated at testing position *l*. Similarly let $Û(l)=\overset{n}{\underset{i=1}{\sum }}{Û}_{i}(l)$ and $\hat{V}(l)=\overset{n}{\underset{i=1}{\sum }}{Û}_{i}(l){Û}_{i}^{\prime }(l)$ be evaluations of Û  and $\hat{V}$ at testing position *l*, respectively. Then the steps of the resampling score-based algorithm are: 

1. generate *n* independent normal variables *z*_*i*_ (*i* = 1,2,· · · ,*n*) from N(0,1);

2. for each *l*, compute ${Û}^{*}(l)=\overset{n}{\underset{i=1}{\sum }}{Û}_{i}(l)z_{i}$, $S^{*}(l)={Û}^{*\prime }(l){\hat{V}}^{-1}(l){Û}^{*}(l)$. Then, compute $S^{*}=\underset{l\in \zeta }{\text{max}}\{S^{*}(l)\}$;

3. repeat *steps* 1 and 2 many times, say *N* times (resampling), to obtain a sequence $\left(S_{1}^{*},S_{2}^{*},\cdot \cdot \cdot \;,S_{N}^{*}\right)$;

4. the score-based threshold for a given significance *α*-level is the 100(1 − *α*) percentile of the ascending ordered values $\left(S_{\left(1\right)}^{*},S_{\left(2\right)}^{*},\cdot \cdot \cdot \;,S_{\left(N\right)}^{*}\right)$.

If ${Û}_{i}(l)$ in ${Û}^{*}(l)$ and $\hat{V}(l)$ are assumed to be fixed and *z*_*i*_ in ${Û}^{*}(l)$ to be random, then: (**I**) The conditional distribution of ${Û}^{*}(l)$ on the observed data is normal with mean zero and limiting covariance as that of Û(l); (**II**) From **I**, it follows that the distributions of $n^{-\frac{1}{2}}{Û}^{*}(l)$ and $n^{-\frac{1}{2}}Û(l)$ are asymptotically equivalent; and, (**III**) From **II**, it is possible to approximate the distribution of *S*(*l*) by that of *s*^∗^(*l*) under the null hypothesis [17,37].

#### Model selection

The search for QTL effects on phenotypic traits consists on identifying those subset of genomic regions for which statistical tests are significant. [38] elaborated the problem of finding such a subset of genomic regions as the one of model selection, for which many tools are available in the vast literature of variable selection. However, in QTL studies the identification of a reasonable model, which maximizes the correct number of QTL while controlling the rate of false discovery is predominant over the identification of models with the smallest prediction errors, which is the major criterion for model selection [38].

The score-based threshold can be used as a criterion to build and refine models with many QTL. Starting with a model with no QTL effect we can select putative QTL and refine the model, by including to or excluding from the MTMIM model any effects, all based on their statistical significance assessed via the score-based method. We propose an algorithm, analogue to the algorithm described in [11], to build an initial MTMIM model and to refine it upon using the score-based threshold criterion.

#### Forward selection

Assuming that model (1) starts with no QTL, one QTL is added at each step of the forward selection. In the *m*^*t*ℎ^ step of the forward selection, we assume a putative pleiotropic QTL at every position *l* ∈ ***ζ*** (one at the time), but avoiding positions within 5 cM neighboring regions of the *m* − 1 QTL already in the model and compute the MLE of all parameters. For each position *l*, we compute the LRT statistic to test the null hypothesis ${\text{H}}_{0}:{\left(\beta_{1m},\beta_{2m},\cdot \cdot \cdot \;,\beta_{\mathrm{Tm}}\right)}^{\prime }={\left(0,0,\cdot \cdot \cdot \;,0\right)}^{\prime }$ versus ${\text{H}}_{1}:{\left(\beta_{1m},\beta_{2m},\cdot \cdot \cdot \;,\beta_{\mathrm{Tm}}\right)}^{\prime }\neq {\left(0,0,\cdot \cdot \cdot \;,0\right)}^{\prime }$. A putative QTL at the position with maximum LRT statistic is added to the model if the LRT statistic is larger than the score-based threshold. Next, the effect of the selected QTL on each trait is tested individually against the null of no effect using the LRT and critical value from a chi-squared probability distribution function with one degree of freedom and pre-specified corrected error rate *α*_*c*_, i.e., when *T* traits are analyzed jointly, the corrected significance level (Bonferroni correction) to test each effect of the *m*^*t*ℎ^ QTL at an error rate *α* is *α*_*c*_ = *α* / *T*. Finally, any nonsignificant effect of the *m*^*t*ℎ^ QTL is removed from the model, ending the *m*^*t*ℎ^ step of the forward selection. The forward selection continues until no maximum LRT statistic exceeds the score-based threshold.

#### Model optimization

In turns, we update the positions of all QTL in the model. We pick a QTL and hold the other QTL fixed at the positions that they were mapped. The effects of the picked QTL are then removed from the model and a new search is done within the region delimited by its two neighboring QTL, avoiding 5 cM from each neighbor (the search is performed until the end of the chromosome if no neighbor QTL is found on either side of the picked QTL). The new position of the picked QTL is set to the position of the maximum LRT statistic within the searched region and all parameters in the model are updated. This procedure is repeated until the positions of all QTL are updated.

### Some suitable hypotheses in the MTMIM model

#### Testing pleiotropic versus closely linked nonpleiotropic QTL

Although testing for pleiotropic versus closely linked nonpleiotropic QTL is a part of model selection, we preferred to separate it from the model selection because in general this test is performed at the end of the model selection procedure, when the final model is almost fitted.

As previously stated, an advantage of multiple trait analysis is the possibility of testing for a single locus affecting multiple traits versus the alternative of two or more closely linked nonpleiotropic loci. For instance, suppose we have measurements of two traits and a total of three nonepistatic QTL at positions *λ*_1_, *λ*_2_ and *λ*_3_. The multiple trait multiple QTL pleiotropic model for a subject *i* would look like:

(5) $$\left(\begin{array}{c} y_{1i}\\ y_{2i} \end{array}\;\right)=\left(\begin{array}{c} u_{1}\\ u_{2} \end{array}\;\right)+\left(\begin{array}{c} \beta_{11}\;\;\beta_{12}\;\;\beta_{13}\\ \beta_{21}\;\;\beta_{22}\;\;\beta_{23} \end{array}\;,\;\right)\;\left(\begin{array}{c} x_{i1}\\ x_{i2}\\ x_{i3} \end{array}\;\right)+\left(\begin{array}{c} e_{1i}\\ e_{2i} \end{array}\;\right)$$

The model above assumes that all QTL have the same pattern of pleiotropy, but instead, suppose we want to test whether the last locus in model (5) is indeed two closely linked nonpleiotropic loci. The model with two pleiotropic (positions *λ*_1_ and *λ*_2_) and two closely linked nonpleiotropic QTL (positions *λ*_3_ and *λ*_4_) for a subject *i* would look like:

(6) $$\left(\begin{array}{c} y_{1i}\\ y_{2i} \end{array}\;\right)=\left(\begin{array}{c} u_{1}\\ u_{2} \end{array}\;\right)+\left(\begin{array}{c} \beta_{11}\;\;\beta_{12}\;\;\beta_{13}\;\;\;0\\ \beta_{21}\;\;\beta_{22}\;\;\;\;0\;\;\beta_{24} \end{array}\;\right)\;\left(\begin{array}{c} x_{i1}\\ x_{i2}\\ x_{i3}\\ x_{i4} \end{array}\;\right)+\left(\begin{array}{c} e_{1i}\\ e_{2i} \end{array}\;\right)$$

Or, suppose we want to test whether the last two QTL in the model (6) are both pleiotropic. The model with four pleiotropic QTL for a subject *i* would look like:

(7) $$\left(\begin{array}{c} y_{1i}\\ y_{2i} \end{array}\;\right)=\left(\begin{array}{c} u_{1}\\ u_{2} \end{array}\;\right)+\left(\begin{array}{c} \beta_{11}\;\;\beta_{12}\;\;\beta_{13}\;\;\beta_{14}\\ \beta_{21}\;\;\beta_{22}\;\;\beta_{23}\;\;\beta_{24} \end{array}\;\right)\;\left(\begin{array}{c} x_{i1}\\ x_{i2}\\ x_{i3}\\ x_{i4} \end{array}\;\right)+\left(\begin{array}{c} e_{1i}\\ e_{2i} \end{array}\;\right)$$

Many hypotheses can be formulated and tested, for example, the hypotheses of model (5) versus (6) can be stated as *H*_0_ : *λ*_3_ = *λ*_4_ versus *H*_1_ : *λ*_3_ ≠ *λ*_4_, and the hypotheses of model (6) versus (7) can be stated as *H*_0_ : *β*_14_ = *β*_23_ = 0 versus *H*_1_ : *β*_14_ ≠ 0 and *β*_23_ ≠ 0. In general, testing whether QTL *r* has pleiotropic main effect or not in a subset *S* (*S* ∈ *T*) of traits in the model, means testing *H*_0_ : *β*_*tr*_ = 0 ∀ *T* ∈ *s* versus *H*_1_ : *β*_*tr*_ ≠ 0 for some *T* ∈ *s*. And, testing whether QTL *r* and *l* has pleiotropic epistatic effect or not in a subset *S* (*S* ∈ *T*) of traits in the model, means testing *H*_0_ : *w*_*trl*_ = 0 ∀ *T* ∈ *s* versus *H*_1_ : *w*_*trl*_ ≠ 0 for some *T* ∈ *s*. Model (6) illustrates a situation in which parameters are constrained to zero and the parameter estimators derived previously in the *CM-step with constrained parameters* are suitable.

When models are nested, the critical value to assess the strength of the LRT is straightforward, in the sense that under regular conditions the LRT has asymptotic chi-squared distribution with degrees of freedom equal to the difference between the number of parameters in the full and reduced models. However, the pleiotropic and closely linkage models may not be nested (for instance, models (6) and (7)), which then requires some correction for the LRT [39,40]. The parametric bootstrap method [13] is an alternative for computing the empirical distribution of the LRT statistic in QTL mapping when models are not nested. In recognizing the test of pleiotropic versus closely linked nonpleiotropic QTL as one of model selection, we evaluate the performance of Akaike’s Information Criterion corrected (AICc) [41] and LRT, using simulation.

When a QTL has epistasis, testing this QTL for pleiotropy versus close linkage is not trivial because the test not only depends on the QTL being tested but also on any other QTL in the model that might interact with it. In general, we suggest to search for QTL main effects, and upon finishing this search to test for pleiotropy versus close linkage, and finally to search for epistasis and no longer to test pleiotropy or to test solely those QTL without epistasis.

#### QTL by environment interaction

The possibility of testing for QTL by environment interaction arises as another advantage of the multiple trait analysis. There are two situations in which we are able to study the differential expression of QTL. First, when the same set of genotypes are evaluated phenotypically in different environments (design I), and second when the phenotypic evaluations are done in different sets of genotypes in different environments (design II) [10]. We regard the model for analysis of data in design II as multiple population model, and thus we shall omit further discussion about it while talking about the multiple trait analysis in this paper.

Let us reiterate that in design I we regard the expression of a trait in different environments as different trait states [42]. Therefore, the index *T* (*T* = 1,2,· · · *T*), which was previously defined to index traits, is regarded as the environment index in what follows. With this in mind, testing whether the main effect of QTL *r* on a trait is statistically different or not in a subset *S* (*S* ∈ *T*) of environments, means testing *H*_0_ : *β*_*tr*_ = *β*_*r*_ ∀ *T* ∈ *s* versus *H*_1_ : *β*_*tr*_ ≠ *β*_*r*_ for some *T* ∈ *s*. And, testing whether QTL *r* and *l* epistatic effect on a trait is statistically different or not in a subset *S* (*S* ∈ *T*) of environments, means testing *H*_0_ : *w*_*trl*_ = 0 ∀ *T* ∈ *s* versus *H*_1_ : *w*_*trl*_ ≠ 0 for some *T* ∈ *s*.

The LRT may be used to evaluate the hypotheses above. The cut-off point for the test can be obtained from the chi-squared probability distribution function with degrees of freedom being the difference between the number of parameters in the full (H_1_) and reduced (H_0_) models.

### Evaluation of the MTMIM model by simulation

We implemented the MTMIM model and score-based threshold method, and evaluated them with several simulated datasets. More specifically, we evaluated type I error, model fitting, and the efficiency of pleiotropic versus closely linked nonpleiotropic QTL testing hypothesis delivered by the MTMIM model.

#### Genome-wide type I error

We use simulation to evaluate the proportion of falsely discovered QTL (type I error) in the analysis of datasets simulated without QTL effects. The LRT statistic is used for hypothesis testing and the score-based threshold is used as the criterion to assess significance level of QTL effects in a genome-wide scan. Each replicate has six chromosomes, each with nine markers evenly spaced 10 cM apart from each other, 300 subjects, and three quantitative traits (see Scenario S0 in Table 8). In the genome-wide scan a putative pleiotropic QTL with main effects on all traits, $\beta ={\left(\beta_{1},\beta_{2},\beta_{3}\right)}^{\prime }$, was assumed at each 1 cM in the genome as the alternative hypothesis. The effects of putative QTL were then tested against the simulated null hypothesis of no effects, $\beta ={\left(\beta_{1},\beta_{2},\beta_{3}\right)}^{\prime }={\left(0,0,0\right)}^{\prime }$ (Scenario S0 of Table 8). For each position in the genome, we resampled the score statistic 1000 times to obtain the genome-wide score-based threshold. One thousand replicates were analyzed in this type I error study.

**Table 8:** Simulated genetic architecture of traits

				**Effects of each QTL**^***d***^					***∑***_***e***_^***f***^		
**Scenario**^***a***^		**h**^**2**^^***b***^	***u***^***c***^	**Q1**	**Q2**	**Q3**	**Q4**	**Q5**	**T1**	**T2**	**T3**
	T1	0	30	0	0	0	0	0	1	0.2	0
S0	T2	0	35	0	0	0	0	0	0.2	1	-0.2
	T3	0	30	0	0	0	0	0	0	-0.2	1
	T1	25	30	0.52	0.52	0.52	0.52	0.52	1	0.2	0
	T2	25	35	0.52	0.52	0.52	0.52	0.52	0.2	1	-0.2
SI	T3	25	30	0.52	0.52	0.52	0.52	0.52	0	-0.2	1
	Chr.	–	–	1	2	3	5	6	–	–	–
	Position^*e*^	–	–	23	15	45	67	53	–	–	–
	T1	25	30	0.52	0.52	0.52	0.52	0.52	1	0.2	0
	T2	18	35	0	0.54	0.54	0.54	0	0.2	1	-0.2
SII	T3	5	30	0	0	0.46	0	0	0	-0.2	1
	Chr.	–	–	1	2	3	5	6	–	–	–
	Position	–	–	23	15	45	67	53	–	–	–
	T1	18	30	0.54	0	0.54	0	0.54	1	0.2	–
	T2	18	35	0	0.54	0.54	0.54	0	0.2	1	–
SIII	Chr.	–	–	1	1	3	6	6	–	–	–
	Position	–	–	23	33	45	38	53	–	–	–

Simulated genetic architecture of traits T1, T2, and T3, as dictated by QTL Q1, Q2, Q3, Q4, and Q5.

^*a*^ Scenario S0 is for type I error evaluation. Scenarios SI, SII and SIII are for model fitting evaluations.

^*b*^ Heritability (%) due to all QTL affecting a trait.

^*c*^ General mean of each trait.

^*d*^ Main effect of QTL. The percentage of phenotypic variation of each trait due to each QTL is 5%.

^*e*^ Position, in cM, of the QTL from the leftmost marker in the chromosome (Chr).

^*f *^ Residual variance-covariance matrix.

#### Model fit evaluations

We use simulation to evaluate the overall performance of the MTMIM model and score-based threshold as the criterion to assess the significance level of QTL effects in the genome-wide scan. We examined the performance of the MTMIM in three different scenarios (SI, SII and SIII shown in Table 8), each evaluated with *r* = 500 replicates. Each replicate was simulated with six chromosomes, each with nine markers evenly spaced 10 cM apart from each other, and 300 subjects. The genetic architecture of quantitative traits in each scenario is described with details in Table 8. For each replicate we build an MTMIM model using our proposed forward selection and model optimization procedure. The genome was partitioned at 1-cM grid for genome-wide scan. For the sake of comparison, we also build an MIM model for each trait in each replicate using our proposed forward selection and model optimization procedure. For every position in the genome, the score statistic was resampled 800 times for the purpose of genome-wide score-based threshold estimation.

The general goal of each simulated scenario is: (SI) With a basic and favorable situation, we want to evaluate basic properties of the MTMIM model; (SII) With a mixture of QTL affecting one, two and three traits, we want to evaluate how well the MTMIM model handles the estimation of QTL with effects on only a subset of traits; (SIII) With presence of closely linked nonpleiotropic QTL and a pleiotropic QTL, we want to evaluate the MTMIM model under more complex genetic architecture. In SIII, we build an MTMIM model for each replicate using the forward selection without testing for pleiotropic versus closely linked nonpleiotropic QTL. Each MTMIM model built in the forward selection was then refined with a follow-up test of pleiotropic versus closely linked nonpleiotropic QTL. The pleiotropic versus closely linked nonpleiotropic test was carried out for every pleiotropic QTL in the MTMIM model.

We evaluated the MTMIM model under three genome-wide significance levels: 1, 5 and 10%. For each replicate, all QTL selected in the forward selection are defined as **mapped** QTL. We summarize the performance of the MTMIM model with measures that are function of the logarithm of odds ratio (LOD) support interval of mapped QTL. The LOD-*d* (*d* = 1, 1.5, and 2) support interval of a mapped QTL is a continuous genomic region that includes the position of the mapped QTL and all positions on its left and right sides with LOD values greater than or equal to the LOD value at the position of the mapped QTL after subtraction of a positive constant *d*[1]. Let *Q*_*r*_, for *r* ∈ {1, 2, · · · *m* = 5}, be a simulated QTL. A simulated QTL is defined as being **paired** with a mapped QTL if the simulated and mapped QTL are nearby. A mapped QTL is defined as being **matched** to a paired QTL if the LOD-*d* support interval of the mapped QTL includes the paired QTL. A mapped QTL is defined as **mismatched** if it is not matched. A simulated QTL *Q*_*r*_ is defined as **identified** if it has a matched QTL. For each simulated *Q*_*r*_ and for each *d*, let $\Omega_{Q_{r},d}$ be the set of replicates for which *Q*_*r*_ is identified. We define $\left|\Omega_{Q_{r},d}\right|$ as the number of elements in $\Omega_{Q_{r},d}$. A criterion to match mapped and simulated QTL which uses both LOD-*d* support interval and closest distance between mapped and simulated QTL is more appropriate than the usual criterion that uses closest distance alone. Our measures of model fit are: (1) False discovery rate per replicate, FDR_*b*_(*d*), which is the ratio of number of mismatched QTL in replicate *b* to total number of mapped QTL in replicate *b*; (2) *FDR* over all replicates, FDR(*d*)= $\overset{R}{\underset{b=1}{\sum }}\mathrm{FD}R_{b}(d)/R$; (3) *Power* to identify *Q*_*r*_, Power(*Q*_*r*_*d*)= $|\Omega_{Q_{r},d}|/R$; (4) *LOD-d support interval coverage* of *Q*_*r*_, *c*(*Q*_*r*_*d*), which is the ratio of $\left|\Omega_{Q_{r},d}\right|$ to the number of replicates for which *Q*_*r*_ is paired with a mapped QTL; (5) *Mean length of LOD-d support interval* of *Q*_*r*_, which is the average length of LOD-*d* support intervals of *Q*_*r*_ over replicates in $\Omega_{Q_{r},d}$; (6) *Mean effect* of *Q*_*r*_, which is the average effects of *Q*_*r*_ over replicates in $\Omega_{Q_{r},d}$; (7) *Mean position* of *Q*_*r*_, which is the average positions of *Q*_*r*_ over replicates in $\Omega_{Q_{r},d}$; and (8) *Model size*, which is the number of mapped QTL. These summary statistics have been proposed by C. Laurie, S. Wang, L. A. Carlini-Garcia and Z-B. Zeng (unpublished observations).

## Appendix

### Parameter estimation

#### Expectation-conditional maximization algorithm

Let $z_{i}^{*}={\left(z_{i1}^{*},z_{i2}^{*},\cdot \cdot \cdot \;,z_{i2^{m}}^{*}\right)}^{\prime }$ be a vector with information on “missing” genotypes of *m* QTL for subject *i*. Each $z_{\mathrm{ij}}^{*}=1$ if *i*^*t*ℎ^ subject has genotype *G*_*j*_ (j = 1,2,· · ·,2^*m*^ ), otherwise $z_{\mathrm{ij}}^{*}=0$. Let $z^{*}=(z_{1}^{*},z_{2}^{*},\cdot \cdot \cdot \;,z_{n}^{*})$ be a matrix containing missing information from all subjects. The joint distribution of observed and missing data $\left(y_{i},z_{i}^{*}\right)$ for subject *i* is:

$$p\left(y_{i},z_{i}^{*}\right)=\overset{2^{m}}{\underset{j=1}{\prod }}{\left[\phi (y_{i}|u+BZ_{\left[\cdot ,j\right]},\sum_{e})p_{\mathrm{ij}}\right]}^{z_{\mathrm{ij}}^{*}}$$

where $p_{\mathrm{ij}}=P(G_{j}|M_{i},R,\lambda )$, and $\phi (y_{i}|u+BZ_{\left[\cdot ,j\right]},\sum_{e})$ is the probability density distribution of a multivariate normal random vector *y*_*i*_ with mean vector $u+BZ_{\left[\cdot ,j\right]}$ and variance-covariance matrix ***∑***_*e*_ . The joint distribution of observed and missing data allow us to obtain the complete-data logarithm likelihood function (*ℓ*_*c*_):

$$\begin{array}{cc} \;\ell_{c}\left(\theta |Y,z^{*}\right) & =\overset{n}{\underset{i=1}{\sum }}\overset{2^{m}}{\underset{j=1}{\sum }}z_{\mathrm{ij}}^{*}\left(\log p_{\mathrm{ij}}+\log\right)\\ & \;\;\;\;\times \left(\left(\phi \left(y_{i}|u+BZ_{\left[\cdot ,j\right]},\sum_{e}\right)\right)\right) \end{array}$$

The E-step requires computation of the expectation of the complete-data logarithm likelihood function, conditional on the observed data *y* and evaluated at current estimated values of ***θ*** (denoted here as ***θ***^(*v*)^ ) [32]:

$$\begin{array}{cc} \;Q_{c}\left(\theta |\theta^{\left(\nu \right)}\right) & =E_{\theta =\theta^{\left(\nu \right)}}\left[\ell_{c}\left(\theta |y,z^{*}\right)|y\right]\\ & =\overset{n}{\underset{i=1}{\sum }}\overset{2^{m}}{\underset{j=1}{\sum }}\Pi_{\mathrm{ij}}^{\left(\nu \right)}\left(\log p_{\mathrm{ij}}+\log\right)\\ & \;\;\;\;\;\;\times \left(\left(\phi \left(y_{i}|u+BZ_{\left[\cdot ,j\right]},\sum_{e}\right)\right)\right) \end{array}$$

where

$$\begin{array}{cc} \;\Pi_{\mathrm{ij}}^{\left(\nu \right)} & =E_{\theta =\theta^{\left(\nu \right)}}\left[z_{\mathrm{ij}}^{*}|y_{i}\right]\\ & =\frac{p_{\mathrm{ij}}\phi \left(y_{i}|u^{\left(\nu \right)}+B^{\left(\nu \right)}Z_{\left[\cdot ,j\right]},\sum_{e}^{\left(\nu \right)}\right)}{\overset{2^{m}}{\underset{j=1}{\sum }}p_{\mathrm{ij}}\phi \left(y_{i}|u^{\left(\nu \right)}+B^{\left(\nu \right)}Z_{\left[\cdot ,j\right]},\sum_{e}^{\left(\nu \right)}\right)} \end{array}$$

The CM-step consists of maximizing the expected complete logarithm likelihood function with respect to the unknown parameters through derivatives (see Section Derivatives).

#### Newton-Raphson method

The NR updating formula for parameter estimation [32] is:

(8) $$\theta^{\left(\nu +1\right)}=\theta^{\left(\nu \right)}+{\left({\left(-\frac{\partial^{2}\ell (\theta |Y)}{\partial \theta \partial \theta^{\prime }}\right|}_{\theta^{\left(\nu \right)}}\right)}^{-1}{\left(\frac{\partial \ell (\theta |Y)}{\partial \theta }\right|}_{\theta^{\left(\nu \right)}}$$

The NR method is not very stable for complex functions because it requires accurate initial values of parameters, in certain problems, in order for right convergency. Moreover, the NR method has almost equally chances to move either in the direction of saddle points, local minima or local maxima [32]. Nevertheless, NR method has a major advantage in terms of quadratic convergence rate (when it does converge) and it can provide an estimate of the variance-covariance matrix of parameters at the limiting value of ***θ***, ***θ***^∗^, through the inverse of the observed Fisher’s information matrix:

$$I^{-1}\left(\theta^{*}|Y\right)={\left({\left(-\frac{\partial^{2}\ell (\theta |Y)}{\partial \theta \partial \theta^{\prime }}\right|}_{\theta^{*}}\right)}^{-1}$$

#### Generalized EM-Newton-Raphson method

By introducing a step-size *κ*^ (*v*)^ (0 <*κ*^ (*v*)^ ≤ 1) and by having the incomplete-data logarithm likelihood function (*ℓ*) replaced by the expected complete-data logarithm likelihood function (*Q*_*c*_) in the updating NR formula (8), a modified version of the updating equation [32] is:

(9) $$\theta^{\left(\nu +1\right)}=\theta^{\left(\nu \right)}+\kappa^{\left(\nu \right)}\;{\left(\;\;{\left(-\frac{\partial^{2}Q_{c}(\theta |Y)}{\partial \theta \partial \theta^{\prime }}\right|}_{\theta^{\left(\nu \right)}}\;\right)}^{-1}\;\;{\left(\frac{\partial Q_{c}(\theta |Y)}{\partial \theta }\right|}_{\theta^{\left(\nu \right)}}$$

The advantage of using the modified version of the updating equation is that an appropriate choice of *κ*^(*v*)^ guarantees that the logarithm likelihood function increases at each iteration. The negative of the matrix of second order derivatives is positive definite under usual conditions. Therefore, its inverse has the Cholesky decomposition (10), where ***c*** is an upper triangular matrix.

(10) $${\left({\left(-\frac{\partial^{2}Q_{c}(\theta |Y)}{\partial \theta \partial \theta^{\prime }}\right|}_{\theta^{\left(\nu \right)}}\right)}^{-1}=C^{\prime }C$$

Let ***θ***^(*ξ*)^ be a point in the line segment from ***θ***^(*v*)^ to ***θ***^(*v* + 1)^, the Taylor’s expansion of the complete-data logarithm likelihood function around ***θ***^(*v*)^ is:

(11) $$\;\begin{array}{cc} Q_{c}\;\left(\;\theta^{\left(\nu +1\right)}|Y\;\right)-Q_{c}\;\left(\;\theta^{\left(\nu \right)}|Y\;\right)\; & \;={\left(\;\theta^{\left(\nu +1\right)}-\theta^{\left(\nu \right)}\;\right)}^{\prime }\;{\left(\frac{\partial Q_{c}(\theta |Y)}{\partial \theta }\;\right|}_{\theta^{\left(\nu \right)}}\\ & \;+\frac{1}{2}\;{\left(\;\theta^{\left(\nu +1\right)}-\theta^{\left(\nu \right)}\;\right)}^{\prime }\;\;{\left(\frac{\partial^{2}Q_{c}(\theta |y)}{\partial \theta \partial \theta^{\prime }}\;\right|}_{\theta^{\left(\xi \right)}}\\ & \;\times \left(\theta^{\left(\nu +1\right)}-\theta^{\left(\nu \right)}\right) \end{array}$$

Plugging ***θ***^(*v*)^ from (2) into (11), and upon making some algebra using (10), we obtain:

(12) $$\begin{array}{c} Q_{c}\;\left(\;\theta^{\left(\nu +1\right)}|Y\;\right)\;-\;Q_{c}(\theta^{\left(\nu \right)}|Y)\;=\;\kappa^{\left(\nu \right)}\;\;{\left(\;{\left(\frac{\partial Q_{c}(\theta |Y)}{\partial \theta }\right|}_{\theta^{\left(\nu \right)}}\right)}^{\prime }\;\;C^{\prime }CB{\left(\frac{\partial Q_{c}(\theta |Y)}{\partial \theta }\right|}_{\theta^{\left(\nu \right)}} \end{array}$$

where

(13) $$B=\left(I+\frac{1}{2}\kappa^{\left(\nu \right)}{\left(\frac{\partial^{2}Q_{c}(\theta |Y)}{\partial \theta \partial \theta^{\prime }}\right|}_{\theta^{\left(\xi \right)}}C^{\prime }C\right)$$

and ***I*** is an identity matrix. From (12), we can see that so long as *κ*^(*v*)^ is chosen to make (13) positive definite, the logarithm likelihood function is guaranteed to increase at every iteration.

### Derivatives

We provide analytical formulae of the first and second order derivatives of the logarithm of individual and overall likelihood functions of data under the MTMIM model. We borrowed useful ideas from [43,44]. These papers provide many results regarding matrix derivatives as well as their applications in multivariate analysis.

#### Auxiliary matrices

We assume *b* = 1, 2, · · · , *s*, *i* = 1, 2, · · · ,*n* and *j* = 1, 2, · · ·  ,2^*m*^ .

***J***_*uℓ*_ is a *T* × *T*  matrix with 1 at positions ***J***_[*u*,*ℓ*]_ and ***J***_[*ℓ*,*u*]_, and zero elsewhere ***I*** is a *T* × *T* identity matrix

$$\begin{array}{cc} \;T_{\mathrm{ij}} & =y_{i}-u-BZ_{\left[\cdot ,j\right]}\\ \;S_{\mathrm{ij}} & =\frac{1}{2}T_{\mathrm{ij}}T_{\mathrm{ij}}^{\prime }\sum_{e}^{-1}-\frac{1}{2}I\\ \;\frac{\partial \Pi_{\mathrm{ij}}}{\partial \theta_{b}} & =\Pi_{\mathrm{ij}}\sum_{e}^{-1}\left(T_{\mathrm{ij}}Z_{\left[b,j\right]}-\overset{2^{m}}{\underset{u=1}{\sum }}\Pi_{\mathrm{iu}}T_{\mathrm{iu}}Z_{\left[b,u\right]}\right)\\ \;\frac{\partial \Pi_{\mathrm{ij}}}{\partial u} & =\Pi_{\mathrm{ij}}\sum_{e}^{-1}\left(T_{\mathrm{ij}}-\overset{2^{m}}{\underset{u=1}{\sum }}\Pi_{\mathrm{iu}}T_{\mathrm{iu}}\right)\\ \;\frac{\partial \Pi_{\mathrm{ij}}}{\partial \sum_{e}} & =\Pi_{\mathrm{ij}}\sum_{e}^{-1}\left(S_{\mathrm{ij}}-\overset{2^{m}}{\underset{u=1}{\sum }}\Pi_{\mathrm{iu}}S_{\mathrm{iu}}\right)\\ \;\frac{\partial T_{\mathrm{ij}}}{\partial \theta_{b}^{\prime }} & =-Z_{\left[b,j\right]}I\\ \;\frac{\partial T_{\mathrm{ij}}}{\partial u^{\prime }} & =-I \end{array}$$

$$\begin{array}{cc} \;\frac{\partial \phi \left(y_{i}|u+BZ_{\left[\cdot ,j\right]},\sum_{e}\right)}{\partial \theta_{b}} & =\phi \left(y_{i}|u+BZ_{\left[\cdot ,j\right]},\right)\\ & \;\left(\sum_{e}\right)\sum_{e}^{-1}T_{\mathrm{ij}}Z_{\left[b,j\right]}\\ \;\frac{\partial \phi \left(y_{i}|u+BZ_{\left[\cdot ,j\right]},\sum_{e}\right)}{\partial u} & =\phi \;\left(y_{i}|u\;+\;BZ_{\left[\cdot ,j\right]},\sum_{e}\right)\;\sum_{e}^{-1}\;T_{\mathrm{ij}}\\ \;\frac{\partial \phi \left(y_{i}|u+BZ_{\left[\cdot ,j\right]},\sum_{e}\right)}{\partial \sum_{e}} & =\phi \left(y_{i}|u\;+\;BZ_{\left[\cdot ,j\right]},\sum_{e}\right)\;\sum_{e}^{-1}\;S_{\mathrm{ij}} \end{array}$$

#### First order derivatives of the logarithm of the individual likelihood function

In the following equations we use a short-hand notation *ℓ*_*i*_(***θ***) = *ℓ*_*i*_(***θ***|*y*_*i*_, *M*_*i*_, ***λ***), and assume *b* = 1, 2, · · · ,*s*.

$$\begin{array}{cc} \;\frac{\partial \ell_{i}(\theta )}{\partial \theta_{b}} & =\sum_{e}^{-1}\overset{2^{m}}{\underset{j=1}{\sum }}\Pi_{\mathrm{ij}}T_{\mathrm{ij}}Z_{\left[b,j\right]}\\ \;\frac{\partial \ell_{i}(\theta )}{\partial u} & =\sum_{e}^{-1}\overset{2^{m}}{\underset{j=1}{\sum }}\Pi_{\mathrm{ij}}T_{\mathrm{ij}}\\ \;\frac{\partial \ell_{i}(\theta )}{\partial \sum_{e}} & =\sum_{e}^{-1}\overset{2^{m}}{\underset{j=1}{\sum }}\Pi_{\mathrm{ij}}S_{\mathrm{ij}} \end{array}$$

#### Second order derivatives of the logarithm of the overall likelihood function

In the following equations we use a short-hand notation *ℓ*(***θ***) = *ℓ*(***θ***|*y*,*M*,***λ***), and assume *b* = 1,2,· · · ,*s*, *k* = 1,2,· · · ,*s*, *u* = 1,2,· · · ,*T*, and *ℓ* = 1,2,· · · ,*T*.

$$\;\begin{array}{cc} \;\frac{\partial^{2}\ell (\theta )}{\partial \theta_{k}\prime \partial \theta_{b}} & =\sum_{e}^{-1}\left(\overset{n}{\underset{i=1}{\sum }}\overset{2^{m}}{\underset{j=1}{\sum }}\Pi_{\mathrm{ij}}Z_{\left[b,j\right]}Z_{\left[k,j\right]}T_{\mathrm{ij}}T_{\mathrm{ij}}^{\prime }\right)\sum_{e}^{-1}\\ & \;-\sum_{e}^{-1}\;\;\left(\;\overset{n}{\underset{i=1}{\sum }}\;\overset{2^{m}}{\underset{j=1}{\sum }}\;\Pi_{\mathrm{ij}}Z_{\left[b,j\right]}T_{\mathrm{ij}}\;\overset{2^{m}}{\underset{c=1}{\sum }}\;\Pi_{\mathrm{ic}}Z_{\left[k,c\right]}T_{\mathrm{ic}}^{\prime }\;\;\right)\;\;\sum_{e}^{-1}\\ & \;-\sum_{e}^{-1}\overset{n}{\underset{i=1}{\sum }}\overset{2^{m}}{\underset{j=1}{\sum }}\Pi_{\mathrm{ij}}Z_{\left[b,j\right]}Z_{\left[k,j\right]} \end{array}$$

$$\;\begin{array}{cc} \;\frac{\partial^{2}\ell (\theta )}{\partial u^{\prime }\partial u} & =\sum_{e}^{-1}\left(\overset{n}{\underset{i=1}{\sum }}\overset{2^{m}}{\underset{j=1}{\sum }}\Pi_{\mathrm{ij}}T_{\mathrm{ij}}T_{\mathrm{ij}}^{\prime }\right)\sum_{e}^{-1}\\ & \;-\sum_{e}^{-1}\left(\overset{n}{\underset{i=1}{\sum }}\overset{2^{m}}{\underset{j=1}{\sum }}\Pi_{\mathrm{ij}}T_{\mathrm{ij}}\overset{2^{m}}{\underset{c=1}{\sum }}\Pi_{\mathrm{ic}}T_{\mathrm{ic}}^{\prime }\right)\sum_{e}^{-1}\\ & \;-n\sum_{e}^{-1} \end{array}$$

$$\;\begin{array}{cc} \;\frac{\partial^{2}\ell (\theta )}{\partial u^{\prime }\partial \theta_{b}} & =\sum_{e}^{-1}\left(\overset{n}{\underset{i=1}{\sum }}\overset{2^{m}}{\underset{j=1}{\sum }}\Pi_{\mathrm{ij}}Z_{\left[b,j\right]}T_{\mathrm{ij}}T_{\mathrm{ij}}^{\prime }\right)\sum_{e}^{-1}\\ & \;-\sum_{e}^{-1}\left(\overset{n}{\underset{i=1}{\sum }}\overset{2^{m}}{\underset{j=1}{\sum }}\Pi_{\mathrm{ij}}Z_{\left[b,j\right]}T_{\mathrm{ij}}\overset{2^{m}}{\underset{c=1}{\sum }}\Pi_{\mathrm{ic}}T_{\mathrm{ic}}^{\prime }\right)\sum_{e}^{-1}\\ & \;-\sum_{e}^{-1}\overset{n}{\underset{i=1}{\sum }}\overset{2^{m}}{\underset{j=1}{\sum }}\Pi_{\mathrm{ij}}Z_{\left[b,j\right]} \end{array}$$

$$\;\begin{array}{cc} \;\frac{\partial^{2}\ell (\theta )}{\partial \sum_{e_{{}_{\left[u,\ell \right]}}}\partial \theta_{b}} & =\sum_{e}^{-1}\overset{n}{\underset{i=1}{\sum }}\overset{2^{m}}{\underset{j=1}{\sum }}\frac{\partial \Pi_{\mathrm{ij}}}{\partial \sum_{e_{{}_{\left[u,\ell \right]}}}}T_{\mathrm{ij}}Z_{\left[b,j\right]}\\ & \;-\sum_{e}^{-1}J_{\mathrm{u\ell}}\sum_{e}^{-1}\overset{n}{\underset{i=1}{\sum }}\overset{2^{m}}{\underset{j=1}{\sum }}\Pi_{\mathrm{ij}}Z_{\left[b,j\right]}T_{\mathrm{ij}} \end{array}$$

$$\;\begin{array}{cc} \;\frac{\partial^{2}\ell (\theta )}{\partial \sum_{e_{{}_{\left[u,\ell \right]}}}\partial u} & =\sum_{e}^{-1}\overset{n}{\underset{i=1}{\sum }}\overset{2^{m}}{\underset{j=1}{\sum }}\frac{\partial \Pi_{\mathrm{ij}}}{\partial \sum_{e_{{}_{\left[u,\ell \right]}}}}T_{\mathrm{ij}}\\ & \;-\sum_{e}^{-1}J_{\mathrm{u\ell}}\sum_{e}^{-1}\overset{n}{\underset{i=1}{\sum }}\overset{2^{m}}{\underset{j=1}{\sum }}\Pi_{\mathrm{ij}}T_{\mathrm{ij}} \end{array}$$

$$\;\begin{array}{cc} \;\frac{\partial^{2}\ell (\theta )}{\partial \sum_{e_{{}_{\left[u,\ell \right]}}}\partial \sum_{e}} & =\sum_{e}^{-1}\overset{n}{\underset{i=1}{\sum }}\overset{2^{m}}{\underset{j=1}{\sum }}\frac{\partial \Pi_{\mathrm{ij}}}{\partial \sum_{e_{{}_{\left[u,\ell \right]}}}}S_{\mathrm{ij}}\\ & \;+\frac{1}{2}n\sum_{e}^{-1}J_{\mathrm{u\ell}}\sum_{e}^{-1}\\ & \;-\frac{1}{2}\sum_{e}^{-1}J_{\mathrm{u\ell}}\sum_{e}^{-1}\;\left(\overset{n}{\underset{i=1}{\sum }}\overset{2^{m}}{\underset{j=1}{\sum }}\Pi_{\mathrm{ij}}T_{\mathrm{ij}}T_{\mathrm{ij}}^{\prime }\right)\;\sum_{e}^{-1}\\ & \;-\frac{1}{2}\sum_{e}^{-1}\;\left(\overset{n}{\underset{i=1}{\sum }}\overset{2^{m}}{\underset{j=1}{\sum }}\Pi_{\mathrm{ij}}T_{\mathrm{ij}}T_{\mathrm{ij}}^{\prime }\right)\;\sum_{e}^{-1}J_{\mathrm{u\ell}}\sum_{e}^{-1} \end{array}$$

#### First and second order derivatives of the expected complete-data logarithm likelihood function

Given current estimated values of $\theta ={\left(\theta_{1},\theta_{2},\cdot \cdot \cdot \;,\theta_{s},u,\text{vect}(\sum_{e})\right)}^{\prime }$, denoted as ***θ***^(*v*)^, the first and second order derivatives of the expected complete-data logarithm likelihood function are shown bellow. We assume *b* = 1, 2,· · · ,*s*, *k* = 1,2,· · · ,*s*, *u* = 1, 2, · · · ,*T* and *ℓ* = 1, 2, · · · ,*T*.

$$\begin{array}{cc} \;\frac{\partial Q_{c}\left(\theta |\theta^{\left(\nu \right)}\right)}{\partial \theta_{b}} & =\sum_{e}^{-1}\overset{n}{\underset{i=1}{\sum }}\overset{2^{m}}{\underset{j=1}{\sum }}\Pi_{\mathrm{ij}}^{\left(\nu \right)}T_{\mathrm{ij}}Z_{\left[b,j\right]}\\ \;\frac{\partial Q_{c}\left(\theta |\theta^{\left(\nu \right)}\right)}{\partial u} & =\sum_{e}^{-1}\overset{n}{\underset{i=1}{\sum }}\overset{2^{m}}{\underset{j=1}{\sum }}\Pi_{\mathrm{ij}}^{\left(\nu \right)}T_{\mathrm{ij}}\\ \;\frac{\partial Q_{c}\left(\theta |\theta^{\left(\nu \right)}\right)}{\partial \sum_{e}} & =\sum_{e}^{-1}\overset{n}{\underset{i=1}{\sum }}\overset{2^{m}}{\underset{j=1}{\sum }}\Pi_{\mathrm{ij}}^{\left(\nu \right)}S_{\mathrm{ij}}\\ \;\frac{\partial^{2}Q_{c}\left(\theta |\theta^{\left(\nu \right)}\right)}{\partial \theta_{k}^{\prime }\partial \theta_{b}} & =-\sum_{e}^{-1}\overset{n}{\underset{i=1}{\sum }}\overset{2^{m}}{\underset{j=1}{\sum }}\Pi_{\mathrm{ij}}^{\left(\nu \right)}Z_{\left[b,j\right]}Z_{\left[k,j\right]} \end{array}$$

$$\begin{array}{cc} \;\frac{\partial^{2}Q_{c}\left(\theta |\theta^{\left(\nu \right)}\right)}{\partial u^{\prime }\partial u} & =-n\sum_{e}^{-1}\\ \;\frac{\partial^{2}Q_{c}\left(\theta |\theta^{\left(\nu \right)}\right)}{\partial u^{\prime }\partial \theta_{b}} & =-\sum_{e}^{-1}\overset{n}{\underset{i=1}{\sum }}\overset{2^{m}}{\underset{j=1}{\sum }}\Pi_{\mathrm{ij}}^{\left(\nu \right)}Z_{\left[b,j\right]}\\ \;\frac{\partial^{2}Q_{c}\left(\theta |\theta^{\left(\nu \right)}\right)}{\partial {\sum_{e}}_{{}_{\left[u,\ell \right]}}\partial \theta_{b}} & =-\sum_{e}^{-1}J_{\mathrm{u\ell}}\sum_{e}^{-1}\overset{n}{\underset{i=1}{\sum }}\overset{2^{m}}{\underset{j=1}{\sum }}\Pi_{\mathrm{ij}}^{\left(\nu \right)}Z_{\left[b,j\right]}T_{\mathrm{ij}}\\ \;\frac{\partial^{2}Q_{c}\left(\theta |\theta^{\left(\nu \right)}\right)}{\partial {\sum_{e}}_{{}_{\left[u,\ell \right]}}\partial u} & =-\sum_{e}^{-1}J_{\mathrm{u\ell}}\sum_{e}^{-1}\overset{n}{\underset{i=1}{\sum }}\overset{2^{m}}{\underset{j=1}{\sum }}\Pi_{\mathrm{ij}}^{\left(\nu \right)}T_{\mathrm{ij}} \end{array}$$

$$\;\begin{array}{cc} \;\frac{\partial^{2}Q_{c}\left(\theta |\theta^{\left(\nu \right)}\right)}{\partial {\sum_{e}}_{{}_{\left[u,\ell \right]}}\partial \sum_{e}} & =\frac{1}{2}n\sum_{e}^{-1}J_{\mathrm{u\ell}}\sum_{e}^{-1}\\ & \;-\frac{1}{2}\sum_{e}^{-1}J_{\mathrm{u\ell}}\sum_{e}^{-1}\;\;\left(\;\overset{n}{\underset{i=1}{\sum }}\;\overset{2^{m}}{\underset{j=1}{\sum }}\;\Pi_{\mathrm{ij}}^{\left(\nu \right)}T_{\mathrm{ij}}T_{\mathrm{ij}}^{\prime }\;\right)\;\;\sum_{e}^{-1}\\ & \;-\frac{1}{2}\sum_{e}^{-1}\;\;\left(\;\overset{n}{\underset{i=1}{\sum }}\;\overset{2^{m}}{\underset{j=1}{\sum }}\;\Pi_{\mathrm{ij}}^{\left(\nu \right)}T_{\mathrm{ij}}T_{\mathrm{ij}}^{\prime }\;\right)\;\;\sum_{e}^{-1}J_{\mathrm{u\ell}}\sum_{e}^{-1} \end{array}$$

### Extension to other crosses

The extension of score statistic to other cross types (for instance, intercross F_2_, recombinant inbred lines, double haploids) is straightforward, in fact, the auxiliary matrices, expressions of first and second order derivatives of the logarithm of individual and overall likelihood functions can be straightly obtained from the general expressions derived previously. For a specific cross type, the extension consists basically of building an appropriate design matrix *Z* and matrix of parameters B , and substituting 2^*m*^ in the summations by the appropriate value according to that cross type (for instance, 3^*m*^ for intercross F_2_).

## Competing interests

The authors declare that they have no competing interests.

## Authors’ contributions

LDCES derived the analytical equations, wrote computer code, carried out the simulations and data analysis, summarized and interpreted the results, and wrote the first draft manuscript. ZBZ provided some initial results of multiple trait analysis, intellectual support criticizing the derivation and implementation of methods, and helped drafting the manuscript. SW implemented the MTMIM method in the Windows QTL Cartographer software. All authors approved this manuscript.

## References

[B1] Lander ES Botstein D Mapping mendelian factors underlying quantitative traits using RFLP linkage maps Genetics 1989 121 185 199 2563713 10.1093/genetics/121.1.185 PMC1203601

[B2] Da Costa E Silva Zeng ZB Current progress on statistical methods for mapping quantitative trait loci from inbred line crosses J Biopharmaceutical Stat 2010 20 2 454 481 10.1080/10543400903572845 20309768

[B3] Zeng ZB Theoretical basis for separation of multiple linked gene effects in mapping quantitative trait loci Proc Natl Acad Sci USA 1993 90 23 10972 10976 10.1073/pnas.90.23.10972 8248199 PMC47903

[B4] Zeng ZB Precision mapping of quantitative trait loci Genetics 1994 136 4 1457 1468 8013918 10.1093/genetics/136.4.1457 PMC1205924

[B5] Haley CS Knott SA A simple regression method for mapping quantitative trait loci in line crosses using flanking markers Heredity 1992 69 4 315 324 10.1038/hdy.1992.131 16718932

[B6] Haley CS Knott SA Elsen JM Mapping quantitative trait loci in crosses between outbred lines using least squares Genetics 1994 136 3 1195 1207 8005424 10.1093/genetics/136.3.1195 PMC1205874

[B7] Kao CH Zeng ZB Teasdale RD Multiple interval mapping for quantitative trait loci Genetics 1999 152 3 1203 1216 10388834 10.1093/genetics/152.3.1203 PMC1460657

[B8] Satagopan JM Yandell BS Newton MA Osborn TC A Bayesian approach to detect quantitative trait loci using Markov chain Monte Carlo Genetics 1996 144 2 805 816 8889541 10.1093/genetics/144.2.805 PMC1207571

[B9] Yi N Shriner D Banerjee S Mehta T Pomp D Yandell BS An efficient Bayesian model selection approach for interacting quantitative trait loci models with many effects Genetics 2007 176 3 1865 1877 10.1534/genetics.107.071365 17483424 PMC1931520

[B10] Jiang C Zeng ZB Multiple trait analysis of genetic mapping for quantitative trait loci Genetics 1995 140 3 1111 1127 7672582 10.1093/genetics/140.3.1111 PMC1206666

[B11] Zeng ZB Kao CH Basten CJ Estimating the genetic architecture of quantitative traits Genet Res 1999 74 3 279 289 10.1017/S0016672399004255 10689805

[B12] Maia JM Joint analysis of multiple gene expression traits to map expression quantitative trait loci PhD thesis, North Carolina State University 2007

[B13] Knott SA Haley CS Multitrait least squares for quantitative trait loci detection Genetics 2000 156 2 899 911 11014835 10.1093/genetics/156.2.899 PMC1461263

[B14] Liu J Liu Y Liu X Deng HW Bayesian Mapping of Quantitative Trait Loci for Multiple Complex Traits with the Use of Variance Components Am J of Human Genet 2007 81 304 320 10.1086/519495 17668380 PMC1950806

[B15] Banerjee S Yandell BS Yi N Bayesian quantitative trait loci mapping for multiple traits Genetics 2008 179 2275 2289 10.1534/genetics.108.088427 18689903 PMC2516097

[B16] Churchill GA Doerge RW Empirical threshold values for quantitative trait mapping Genetics 1994 138 3 963 971 7851788 10.1093/genetics/138.3.963 PMC1206241

[B17] Zou F Fine JP Hu J Lin DY An efficient resampling method for assessing genome-wide statistical significance in mapping quantitative trait Loci Genetics 2004 168 4 2307 2316 10.1534/genetics.104.031427 15611194 PMC1448705

[B18] Liu J Mercer JM Stam LF Gibson GC Zeng ZB Laurie CC Genetic analysis of a morphological shape difference in the male genitalia of Drosophila simulans and D. mauritiana Genetics 1996 142 1129 1145 8846893 10.1093/genetics/142.4.1129 PMC1207113

[B19] Zeng ZB Liu J Stam LF Kao CH Mercer JM Laurie CC Genetic architecture of a morphological shape difference between two Drosophila species Genetics 2000 154 299 310 10628989 10.1093/genetics/154.1.299 PMC1460924

[B20] Archive for the genotypic and phenotypic data of the motivating example [ ftp://statgen.ncsu.edu/pub/qtlcart/data/zengetal99/]

[B21] Storey JD Tibshirani R Statistical significance for genomewide studies Proc Natl Acad Sci USA 2003 100 16 9440 9445 10.1073/pnas.1530509100 12883005 PMC170937

[B22] Beavis WD Paterson AH QTL analyses: power, precision, and accuracy Molecular Dissection of Complex Traits 1998 New York: CRC Press 145 162

[B23] Wang S Basten CJ Zeng ZB Windows QTL Cartographer 2.51 Department of Statistics, North Carolina State University, Raleigh, NC, 2011 [ http://statgen.ncsu.edu/qtlcart/WQTLCart.htm]

[B24] Kao CH Zeng ZB Modeling epistasis of quantitative trait loci using Cockerham’s model Genetics 2002 160 3 1243 1261 11901137 10.1093/genetics/160.3.1243 PMC1462022

[B25] Zeng ZB Wang T Zou W Modeling quantitative trait loci and interpretation of models Genetics 2005 169 3 1711 1725 15654105 10.1534/genetics.104.035857 PMC1449562

[B26] Zellner A An efficient method of estimating seemingly unrelated regressions and tests for aggregation bias J Am Stat Assoc 1962 57 298 348 368 10.1080/01621459.1962.10480664

[B27] Yi N Yandell BS Churchill GA Allison DB Eisen EJ Pomp D Bayesian model selection for genome-wide epistatic quantitative trait loci analysis Genetics 2005 170 3 1333 1344 10.1534/genetics.104.040386 15911579 PMC1451197

[B28] Jiang C Zeng ZB Mapping quantitative trait loci with dominant and missing markers in various crosses from two inbred lines Genetica 1997 101 47 58 10.1023/A:1018394410659 9465409

[B29] Dempster AP Laird N Rubin D Maximum likelihood from incomplete data via the EM algorithm J R Stat Soc Ser B (Methodological) 1977 39 1 38

[B30] Rai SN Matthews DE Improving the EM algorithm Biometrics 1993 49 2 587 591 10.2307/2532570

[B31] Aitkin M Aitkin I A hybrid EM/Gauss-Newton algorithm for maximum likelihood in mixture distributions Stat Comput 1996 6 127 130 10.1007/BF00162523

[B32] McLachlan GJ Krishnan T The EM Algorithm and Extensions 1996 New York: Wiley-Interscience

[B33] Meng XL Rubin DB Maximum likelihood estimation via the ECM algorithm: a general framework Biometrika 1993 80 2 267 278 10.1093/biomet/80.2.267

[B34] Redner RA Walker HR Mixiture densities, maximum likelihood and the EM algorithm SIAM Rev 1984 26 2 195 239 10.1137/1026034

[B35] Chang MN Wu R Wu SS Casella G Score statistics for mapping quantitative trait loci Stat Appl Genet Mol Biol 2009 8 1 35 [Article 16] 10.2202/1544-6115.1386 19222383

[B36] Cox DR Hinkley DV Theoretical Statistics 1974 London: Chapman and Hall

[B37] Lin D An efficient Monte Carlo approach to assessing statistical significance in genomic studies Bioinformatics 2005 21 6 781 787 10.1093/bioinformatics/bti053 15454414

[B38] Broman KW Speed TP A model selection approach for the identification of quantitative trait loci in experimental crosses J R Stat Soc Series B (Statl Methodology) 2002 64 4 641 656 10.1111/1467-9868.00354

[B39] Vuong QH Likelihood ratio tests for model selection and non-nested hypotheses Econometrica 1989 57 2 307 333 10.2307/1912557

[B40] Kapetanios G Weeks M Non-nested models and the likelihood ratio statistic: a comparison of simulation and bootstrap based tests Technical report: University of London, 2003

[B41] Sugiura N Further analysts of the data by Akaike’s information criterion and the finite corrections Commun Stat - Theory and Methods 1978 7 13 26 10.1080/03610927808827599

[B42] Falconer DS The problem of environment and selection Am Nat 1952 86 293 298 10.1086/281736

[B43] Dwyer PS Macphail MS Symbolic matrix derivatives Ann Math Stat 1948 19 4 517 534 10.1214/aoms/1177730148

[B44] Dwyer PS Some applications of matrix derivatives in multivariate analysis J Am Stat Assoc 1967 62 318 607 625 10.1080/01621459.1967.10482934

